# Metabolic diversification of nitrogen‐containing metabolites by the expression of a heterologous lysine decarboxylase gene in Arabidopsis

**DOI:** 10.1111/tpj.14454

**Published:** 2019-08-27

**Authors:** Yohei Shimizu, Amit Rai, Yuko Okawa, Hajime Tomatsu, Masaru Sato, Kota Kera, Hideyuki Suzuki, Kazuki Saito, Mami Yamazaki

**Affiliations:** ^1^ Graduate School of Pharmaceutical Sciences Chiba University 1‐8‐1 Inohana, Chuo‐ku Chiba 260‐8675 Japan; ^2^ RIKEN Center for Sustainable Resource Science 1‐7‐22 Suehiro‐cho, Tsurumi‐ku Yokohama 230‐0045 Japan; ^3^ Kazusa DNA Research Institute 2‐6‐7 Kazusa‐Kamatari Kisarazu Chiba 292‐0818 Japan; ^4^ Present address: Human Metabolome Technologies, Inc. 246‐2 Mizukami, Kakuganji Tsuruoka Yamagata 997‐0052 Japan; ^5^ Present address: Department of Biomolecular Engineering Graduate School of Engineering Tohoku University Aobayama 6‐6‐07 Sendai 980‐8579 Japan

**Keywords:** lysine decarboxylase, cadaverine catabolism, *Arabidopsis thaliana*, chemodiversity, non‐targeted metabolome analysis, Lys‐derived alkaloids

## Abstract

Lysine decarboxylase converts l‐lysine to cadaverine as a branching point for the biosynthesis of plant Lys‐derived alkaloids. Although cadaverine contributes towards the biosynthesis of Lys‐derived alkaloids, its catabolism, including metabolic intermediates and the enzymes involved, is not known. Here, we generated transgenic Arabidopsis lines by expressing an exogenous lysine/ornithine decarboxylase gene from *Lupinus angustifolius* (*La‐L/ODC*) and identified cadaverine‐derived metabolites as the products of the emerged biosynthetic pathway. Through untargeted metabolic profiling, we observed the upregulation of polyamine metabolism, phenylpropanoid biosynthesis and the biosynthesis of several Lys‐derived alkaloids in the transgenic lines. Moreover, we found several cadaverine‐derived metabolites specifically detected in the transgenic lines compared with the non‐transformed control. Among these, three specific metabolites were identified and confirmed as 5‐aminopentanal, 5‐aminopentanoate and δ‐valerolactam. Cadaverine catabolism in a representative transgenic line (DC29) was traced by feeding stable isotope‐labeled [α‐^15^N]‐ or [ε‐^15^N]‐l‐lysine. Our results show similar ^15^N incorporation ratios from both isotopomers for the specific metabolite features identified, indicating that these metabolites were synthesized via the symmetric structure of cadaverine. We propose biosynthetic pathways for the metabolites on the basis of metabolite chemistry and enzymes known or identified through catalyzing specific biochemical reactions in this study. Our study shows that this pool of enzymes with promiscuous activities is the driving force for metabolite diversification in plants. Thus, this study not only provides valuable information for understanding the catabolic mechanism of cadaverine but also demonstrates that cadaverine accumulation is one of the factors to expand plant chemodiversity, which may lead to the emergence of Lys‐derived alkaloid biosynthesis.

## Significance Statement

Cadaverine, a reaction product of lysine decarboxylase, serves as an intermediate of plant Lys‐derived alkaloids. Despite being an important metabolite in the expansion of plant chemodiversity, little is known about its catabolism *in planta*. In this study, we identified cadaverine‐derived metabolites and their metabolic pathways in transgenic Arabidopsis, which will provide a molecular basis for understanding the emergence of chemodiversity through cadaverine production.

## Introduction

Plants, being sessile organisms, have an expanded chemodiversity of secondary metabolites to adapt to various environmental conditions (Weng *et al*., 2012; Moghe and Last, 2015; Michael, 2017). As a rough estimate the plant kingdom synthesizes over 1 000 000 metabolites with diverse physiological effects, and several of these have also proven to possess beneficial properties for humans (Saito and Matsuda, 2010; Afendi *et al*., 2012; Yamazaki *et al*., 2018). Since ancient times, therefore, plant metabolite extracts have been used as medicines and as lead compounds to inspire new drug molecules with various pharmacological properties (Kinghorn *et al*., 2011; Rai *et al*., 2017a, 2017b).

The chemodiversity of secondary metabolites has expanded from simple compounds, branched from primary metabolism, through various biochemical reactions, such as oxidation, decarboxylation, condensation, hydroxylation and methylation, among others (Rai *et al*., 2016; Michael, 2017). The branching points of metabolic networks have often been found under strict regulatory control and are keys for the evolution of specialized metabolite biosynthesis in plants (Estévez *et al*., 2001; Glawischnig *et al*., 2004; Reuben *et al*., 2013). For instance, putrescine, an essential metabolite for all living organisms, serves as the branch point for the biosynthesis of different specialized metabolites in plants. The *N*‐methylation of putrescine catalyzed by putrescine *N*‐methyltransferase (PMT) is the branch point of the biosynthesis of ornithine‐derived alkaloids such as nicotine, tropane and nortropane alkaloids (Junker *et al*., 2013; Kajikawa *et al*., 2017). The dimerization of putrescine by homospermidine synthase (HSS) on the other hand triggers the biosynthesis of pyrrolizidine alkaloids, which are distributed in a wide range of plant species (Ober and Hartmann, 1999; Ober *et al*., 2003). PMT and HSS, enzymes at branch points diverting putrescine towards the biosynthesis of secondary metabolites, are highly conserved and have reportedly evolved under positive selection across different plant species (Junker *et al*., 2013; Kaltenegger *et al*., 2013). Therefore, the branching points diverting primary metabolism into secondary metabolism are critical for the emergence of plant chemodiversity.

The conversion of l‐lysine to cadaverine, catalyzed by lysine/ornithine decarboxylase (L/ODC), is the branching point for the biosynthesis of Lys‐derived alkaloids (Bunsupa *et al*., 2012, 2016; Xu *et al*., 2017). L/ODC is a bifunctional enzyme with lysine decarboxylase (LDC) and ornithine decarboxylase (ODC) activities. L/ODC has been shown to have evolved from ancestral ODC, which catalyzes the decarboxylation of l‐ornithine to putrescine (Bunsupa *et al*., 2012, 2016). Evolution towards increased LDC activity in L/ODC has been shown to occur under positive selective pressure for plant species producing Lys‐derived alkaloids (Bunsupa *et al*., 2016), and therefore the evolution of LDC activity to produce cadaverine in plants is considered an important event for the biosynthesis of Lys‐derived alkaloids.

In plants, cadaverine is catabolized by copper‐containing amine oxidase (CuAO) to produce 5‐aminopentanal. 5‐Aminopentanal undergoes further spontaneous cyclization, resulting in Δ^1^‐piperideine, which serves as a universal intermediate of diverse Lys‐derived alkaloids (Braekman *et al*., 1972; Leeper *et al*., 1981; Golebiewski and Spenser, 1985; Sato *et al*., 2018). In addition, cadaverine is also known to be associated with a wide range of plant physiological phenomena, such as growth, development, stress responses and cellular signaling (Smith and Wilshire, 1975; Kuznetsov *et al*., 2007; Tomar *et al*., 2013a, 2013b; Jancewicz *et al*., 2016). Although cadaverine plays important roles in alkaloid biosynthesis and plant physiology, few studies have focused on the detailed metabolism of cadaverine in plants. Furthermore, the mechanism behind the diversion of cadaverine to the biosynthesis of Lys‐derived alkaloids remains unknown. It would be interesting to see whether non‐cadaverine‐producing plants could catabolize cadaverine to increase plant chemodiversity, or even produce alkaloids or alkaloid‐like metabolites, using endogenous enzymes, which would further support the importance of branch points for the evolution of secondary metabolism.

In this study, we established a novel branch pathway from l‐lysine to cadaverine in *Arabidopsis thaliana* by expressing *L/ODC* from *Lupinus angustifolius* (*La‐L/ODC*). *La‐L/ODC*‐expressing transgenic Arabidopsis lines accumulated cadaverine and several of the cadaverine‐derived metabolites, which were not detected in the non‐transformed plant. We further investigated the cadaverine catabolic pathway and its enzymatic components in Arabidopsis. This study will contribute towards understanding the mechanism for the emergence of alkaloid biosynthesis from cadaverine through artificial chemical diversification.

## Results

### Exogenous expression of *La‐L/ODC* resulted in cadaverine accumulation in transgenic Arabidopsis

**Figure 1:**
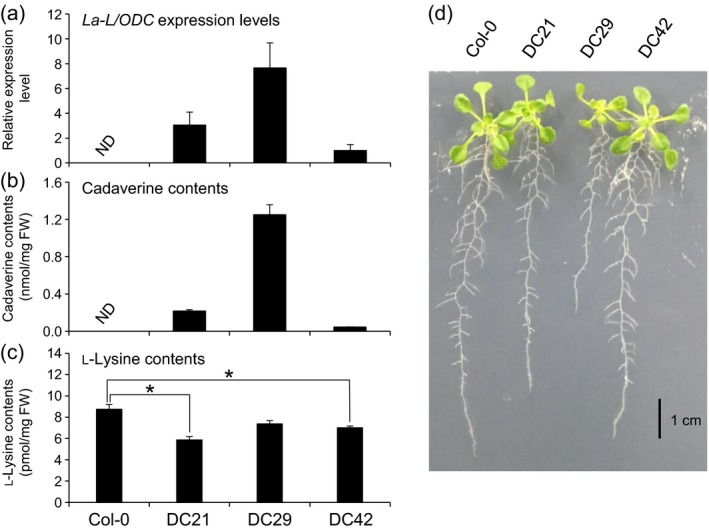
*La‐L/ODC*, cadaverine and l‐lysine levels in DC lines and plant phenotypes. (a) *La‐L/ODC* mRNA levels in 2‐week‐old DC lines determined by quantitative RT‐PCR analysis. Pooled 2‐week‐old seedlings from 30 plants were regarded as a single biological replicate for each line (and for panels b and c). β*‐Tubulin* was used as an internal control. DC42, with the lowest expression level among the three lines, was used to normalize the expression level. Values are means ± standard deviations (*n* = 3). (b) Cadaverine and (c) l‐lysine contents in DC lines. Metabolites were extracted from a pool of 2‐week‐old seedlings and cadaverine/lysine levels were quantified with LC‐MS. Values are means ± standard errors (*n *= 4–6). (d) Phenotypes of 2‐week‐old transgenic plants. *P < 0.05 (Student's *t*‐test); FW, fresh weight; ND, not detected.

To synthesize cadaverine, we generated transgenic Arabidopsis lines (Col‐0 background) expressing *L/ODC* from *L. angustifolius* (*La‐L/ODC*) (Bunsupa *et al*., 2012), a quinolizidine alkaloid‐producing plant. The heterologous *La‐L/ODC* was expressed under the control of the double cauliflower mosaic virus *35S* promoter and the omega enhancer (Figure S1a), and 10 independent T_3_ lines (DC lines) were established. Semi‐quantitative reverse transcription‐PCR (RT‐PCR) for the DC lines showed broad expression levels of *La‐L/ODC*, with DC21, DC29 and DC42 exhibiting the highest expression among all of the lines (Figure S1b). As expected, no expression for *La‐L/ODC* was detected in the non‐transformed Col‐0 plants. We further quantified *La‐L/ODC* expression in the selected DC lines by quantitative RT‐PCR, which showed increased expression levels of 7.7‐ and 3.0‐fold in DC29 and DC21, respectively, when compared with that in DC42 (Figure 1a). We therefore selected DC29 as high, and DC21 and DC42 as moderate, *La‐L/ODC*‐expressing transgenic lines for further characterization.

We next quantified the cadaverine and l‐lysine levels in these DC lines. As expected, cadaverine accumulated in all three DC lines but was not detected in Col‐0 (Figure 1b). The accumulation levels of cadaverine correlated with the *La‐L/ODC* expression levels in the respective DC lines, with cadaverine contents being highest in DC29 (1.25 nmol mg^−1^ fresh weight, FW) followed by DC21 (0.21 nmol mg^−1^ FW) and lowest in DC42 (0.04 nmol mg^−1^ FW). In contrast, l‐lysine contents in DC29, DC21 and DC42 were decreased by 32.9, 15.7 and 19.7%, respectively, compared with the l‐lysine content in Col‐0 (Figure 1c). As La‐L/ODC also catalyzes the decarboxylation of l‐ornithine to form putrescine, we quantified the accumulation levels of l‐ornithine and putrescine (Figure S2). l‐Ornithine levels moderately decreased, whereas the corresponding levels of putrescine significantly increased in all DC lines. The putrescine level was particularly high in DC29, showing an increase of 66.6‐fold when compared with the level found in Col‐0. We next investigated phenotypic changes in DC lines in response to *La‐L/ODC* expression and associated metabolite changes. DC lines showed no apparent morphological change except for DC29, which exhibited a small but statistically significant reduction in root growth and biomass (Figures 1d and S3), similar to results found with the exogenous supplementation of cadaverine (Liu *et al*., 2014; Strohm *et al*., 2015). Taken together, these results show that heterologously expressed La‐L/ODC was functional in Arabidopsis.

### Non‐targeted metabolome profiling captured changes in metabolic processes by *La‐L/ODC* expression

**Figure 2:**
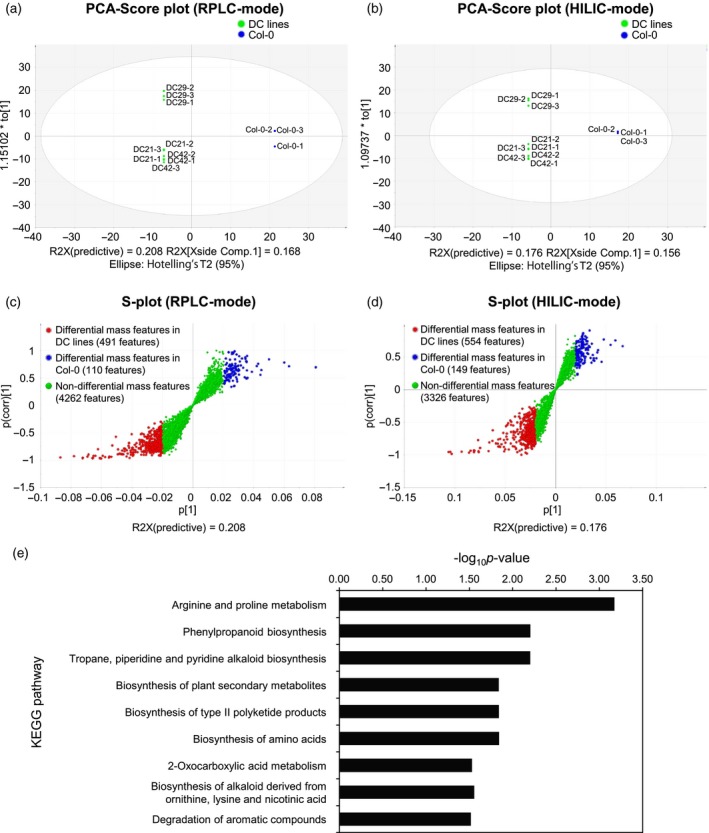
Multivariate analysis of metabolome data and enriched pathways in DC lines. Principal component analysis (PCA) score plot for DC lines (green dots) and Col‐0 (blue dots) in (a) reverse‐phase liquid chromatography (RPLC) mode and (b) hydrophilic interaction chromatography (HILIC) mode. DC lines and Col‐0 were clearly separated along the PC1 axis. Scatter plot of orthogonal partial least‐squares discriminant analysis (OPLS‐DA) model for DC lines versus Col‐0 in (c) RPLC mode and (d) HILIC mode. (e) Pathway enrichment analysis for metabolite peaks associated with DC lines. Differential metabolite features were mapped to the KEGG‐Arabidopsis database and *P* values for each KEGG pathway were calculated by Fisher's exact test. The *P* value cut‐off was 0.05.

Next, we performed non‐targeted metabolite profiling of three DC lines and Col‐0 to assess the global metabolome change brought about by *La‐L/ODC* expression in Arabidopsis. The overall workflow is shown in Figure S4. Non‐targeted metabolome analysis using ultra‐high‐performance liquid chromatography (UHPLC) high‐resolution mass spectrometry extracted 4863 metabolite features in the reverse‐phase liquid chromatography (RPLC) mode (metabolite IDs with RP as the prefix) and 4029 metabolite features in the hydrophilic interaction chromatography (HILIC) mode (metabolite IDs with HI as the prefix) (Tables 1 and S1). A principal component analysis (PCA) score plot of metabolome data showed DC lines and Col‐0 being separated along the PC1 axis (20.8 and 17.6% variations in RPLC and HILIC, respectively), with DC29 and the other two lines separated along the PC2 axis (16.8 and 15.6% variations in RPLC and HILIC, respectively) (Figure 2a,b). These results indicate that metabolite profiling in RPLC and HILIC mode successfully captured Metabo‐phenotypes associated with *La‐L/ODC* expression in Arabidopsis.

**Table 1:** Summary of the number of metabolite features at different stages of metabolome analysis

Analytical stage	RPLC	HILIC	Analysis pipeline
Feature extraction	4863	4029	powerget
Extraction of differential features in DC lines	491	554	OPLS‐DA and S‐plot
Mapping of differential features in DC lines to KEGG database	72	66	PCDL manager and KegArray
Extraction of differential features in Col‐0	110	149	OPLS‐DA and S‐plot
Mapping of differential features in Col‐0 to KEGG database	12	11	PCDL manager and KegArray
Selection of specific peaks in DC lines	46	32	powerget and manual validation
Acquisition of MS/MS spectra of specific peaks	32	29	LC‐MS/MS
MS/MS‐based annotation for specific peaks	24	22	MS‐FINDER
Acquisition of ^15^N‐labeled metabolite features	17	24	powerget and shiftedionsfinder

To identify metabolite features discriminating DC lines from Col‐0, we performed orthogonal partial least‐squares discriminant analysis (OPLS‐DA) using metabolite features as variables, and with DC lines and Col‐0 as two observation classes. OPLS‐DA for the RPLC‐mode data set gave R2X (predictive) and R2X (orthogonal) variations as 0.208 and 0.498, respectively, with a Q2 (cumulative, representing the goodness of prediction and quality of model) value of 0.89 (Figure S5; Table S2). Similarly, OPLS‐DA for the HILIC‐mode data set gave R2X (predictive) and R2X (orthogonal) variations as 0.176 and 0.419, respectively, with a Q2 (cumulative) of 0.842 (Figure S5; Table S2). The high values of Q2 (cumulative) for the OPLS‐DA models using metabolite profiling data in both modes suggest that the metabolite features reliably separated the DC lines and Col‐0 into two groups, and that the models established were suitable to identify correlated metabolite features for each observation group.

An S‐plot, a scatter plot visualizing covariance and correlation between metabolite features and each observation class, offers a powerful means to identify statistically significant metabolites in each group with a lower risk of spurious correlation (Wiklund *et al*., 2008). We used the OPLS‐DA model to derive an S‐plot and identified 491 and 110 differential metabolite features for RPLC mode, and 554 and 149 differential metabolite features for HILIC mode, associated with DC lines and the Col‐0 observation group, respectively (Figure 2c,d; Tables 1 and S3). These differential metabolite features were assigned to a metabolite name, chemical formula and metabolite pathway using the KEGG‐Arabidopsis compound database, resulting in the annotation of 72 (RPLC) and 66 (HILIC) differential metabolite features in DC lines, and 12 (RPLC) and 11 (HILIC) differential metabolites in Col‐0, respectively (Tables 1 and S4). As expected and consistent with our targeted analysis (Figures 1b,c and S2), the differential metabolite features in DC lines included cadaverine (RP_1.63/103.1233 and HI_24/103.1234) and putrescine (HI_24.75/89.1079), whereas the differential features in Col‐0 included l‐lysine (RP_1.7/147.1128 and HI_25.59/147.1128) and l‐ornithine (HI_25.57/133.0973). Furthermore, the differential metabolite features in DC lines included 5‐aminopentanal (RP_2.34/102.0917 and HI_10.3/102.0917), a known catabolite of cadaverine. The conversion of cadaverine into 5‐aminopentanal in DC lines suggests that endogenous enzyme activities diverted cadaverine into downstream metabolites in Arabidopsis.

### Pathway enrichment analysis indicated global metabolic changes in DC lines

Pathway enrichment analysis using differential features in DC lines showed nine KEGG pathways being significantly enriched (Figure 2e). For arginine and proline metabolism (map00330; Figure S6), two biosynthetic intermediates of putrescine from l‐arginine, namely agmatine and *N*‐carbamoylputrescine, and four putrescine‐derived metabolites, including *N*‐acetylputrescine, γ‐l‐glutamylputrescine, *p*‐coumaroylputrescine and spermidine, were significantly accumulated in the DC lines. Metabolite features associated with significantly enriched phenylpropanoid biosynthesis (map00940; Figure S7) included phenylalanine, *p*‐coumaroyl shikimic acid, ferulic acid, sinapic acid, sinapaldehyde, sinapyl alcohol and syringin. Several metabolite intermediates of the phenylpropanoid biosynthetic pathway are used for the conjugation of polyamines such as cadaverine and putrescine, among others, to produce phenolamides in higher plants (Martin‐Tanguy *et al*., 1978; Facchini *et al*., 2002; Muroi *et al*., 2009). The enriched phenylpropanoid biosynthesis in DC lines thus seems to reflect metabolic remodeling through the accumulation of cadaverine and/or putrescine. Interestingly, two alkaloid biosynthetic pathways, namely tropane, piperidine and pyridine alkaloid biosynthesis (map00960; Figure S8) and the biosynthesis of alkaloids derived from ornithine, lysine and nicotinic acid (map01064; Figure S9), were also enriched in DC lines. On the other hand, KEGG pathway enrichment analysis using differential metabolite features in Col‐0 showed no pathways being significantly enriched in Col‐0. With regards to lysine degradation (map00310; Figure S10), however, l‐lysine (RP_1.7/147.1128 and HI_25.59/147.1128) and two known l‐lysine catabolites, namely l‐pipecolate (RP_1.7/130.0864) and l‐saccharopine (RP_24.99/277.1377), significantly increased in Col‐0 but decreased in DC lines. The decreases of these metabolites in DC lines were attributed to a reduction of the l‐lysine pool by the activity of heterologous La‐L/ODC.

### Increased chemodiversity derived from the de novo synthesis of cadaverine in Arabidopsis

Among differentially accumulated metabolite features selected by OPLS‐DA, we obtained 46 and 32 metabolite features in RPLC and HILIC mode, respectively, being specifically accumulated in DC lines but not detected in Col‐0 (Tables 1 and S5). As cadaverine is not synthesized in Arabidopsis (Hanfrey *et al*., 2001), these specific metabolite features in DC lines are strong candidates as cadaverine‐derived metabolites. By data‐dependent MS/MS analysis, we obtained fragmentation data of 32 out of 46 specific metabolite features in RPLC mode, and 29 out of 32 specific metabolite features in HILIC mode (Tables 1 and S5).

Specific metabolite features with MS/MS profiles were then annotated by ms‐finder (Table S5) (Tsugawa *et al*., 2016; Vaniya *et al*., 2017). ms‐finder uses a unique annotation algorithm that considers the hydrogen rearrangement taking place during fragmentation as a result of low energy collision‐induced dissociation and predicts candidate structures from 22 compound databases (with a total of 964 923 compounds in ms‐finder 2.42). Using an ms‐finder‐based approach and comparing MS/MS fragmentation spectra of metabolite features with the fragmentation pattern of compounds deposited in the databases, we annotated 24 and 22 metabolites each in RPLC and HILIC mode, respectively (Tables 1 and S5). As expected and consistent with KEGG‐based annotation, the metabolites identified by MS‐FINDER included cadaverine (RP_1.63/103.1233 and HI_24/103.1234), which was also confirmed with the metabolite standard (Figure S11). Moreover, two cadaverine catabolites, 5‐aminopentanal (RP_2.34/102.0917 and HI_10.3/102.0917) and 5‐aminopentanoate (HI_11.21/118.0865) were also identified. δ‐Valerolactam, a lactam form of 5‐aminopentanoate, was assigned to several metabolite features (*m/z* 100.0761) by ms‐finder‐based analysis through similar fragmentation patterns in RPLC (RP_1.63/100.0761 and RP_9.23/100.0761) and HILIC mode (HI_3.62/100.0761 and HI_11.21/100.0761). ms‐finder annotated several metabolite features as *N*‐acylated cadaverine, including *N*‐acetylcadaverine (RP_2.39/145.1336, RP_3.42/145.1336 and HI_11.43/145.1336) and *p*‐coumaroylcadaverine (HI_6.9/249.1597). Interestingly, MS/MS profiles of several specific metabolite features contained characteristic fragmentation patterns associated with Lys‐derived alkaloids, including slaframine (HI_11.19/199.1442) and (*R*)‐pelletierine (RP_5.12_142.1226 and HI_7.37/142.1228).

**Figure 3:**
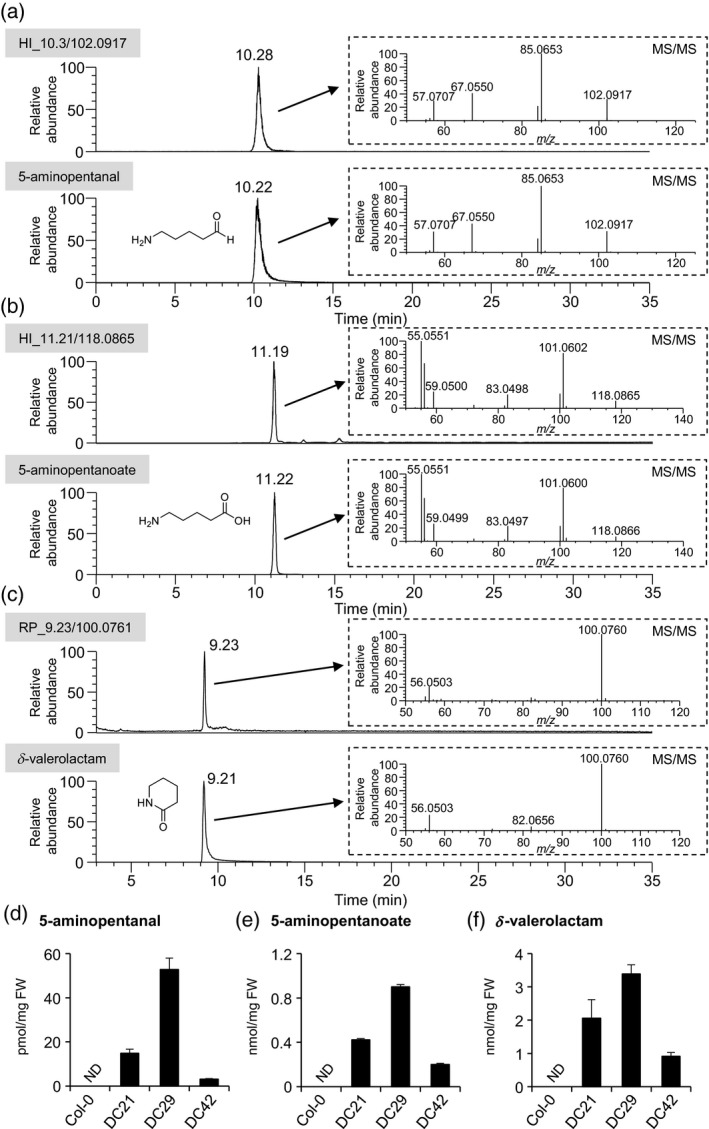
Identification of three specific metabolite features by standard compounds and their accumulation levels among DC lines.The retention times and MS/MS spectra of specific metabolite peaks were compared with those of standards by LC‐MS: (a) 5‐aminopentanal and HI_10.3/102.0917; (b) 5‐aminopentanoate and HI_11.21/118.0865; and (c) δ‐valerolactam and RP_9.23/100.0761. Higher energy collision dissociation (HCD) mode was used to obtain MS/MS fragmentation. Accumulation levels in among DC lines and Col‐0 of: (d) 5‐aminopentanal; (e) 5‐aminopentanoate; and (f) δ‐valerolactam. Metabolites were extracted from pools of 30 seedlings grown for 2 weeks. Single‐ion monitoring mode was used for the quantification of each metabolite. Data are means ± standard errors (*n* = 4–6); FW, fresh weight; ND, not detected.

Among the specific metabolites annotated and confirmed through the ms‐finder‐based approach, we further focused on three metabolites, namely 5‐aminopentanal, 5‐aminopentanoate and δ‐valerolactam, selected based on their beneficial physiological and chemical aspects. By direct comparison with authentic standards using LC‐MS/MS, we identified RP_2.34/102.0917 and HI_10.3/102.0917 as 5‐aminopentanal, HI_11.21/118.0865 as 5‐aminopentanoate, and RP_9.23/100.0761 as δ‐valerolactam, respectively (Figure 3a–c). The accumulation levels of these metabolites identified across DC lines were correlated with *La‐L/ODC* expression in each DC line (Figure 3d–f). These results suggest that synthetically produced cadaverine in Arabidopsis was further converted to several cadaverine‐derived metabolites by endogenous enzymes.

Several studies in the past have shown that biosynthesis enzymes in Arabidopsis, such as AtCuAO3 and AtNATA1, could accept cadaverine as a substrate to produce 5‐aminopentanal and *N*‐acetylcadavrine, respectively. 5‐Aminopentanoate, another specific metabolite for transgenic lines, could be derived from 5‐aminopentanal through a simple dehydrogenation reaction. Previously, two ALDH10 family enzymes, namely AtALDH10A8 and AtALDH10A9, were shown to catalyze the conversion of 4‐aminobutanal to GABA. As 4‐aminobutanal is an analog of 5‐aminobutanal, with one fewer carbon atoms, we asked whether AtALDH10A8 and AtALDH10A9 could also oxidize 5‐aminobutanal. To test this hypothesis, the full open reading frames of each gene were inserted into the pET15b vector containing an N‐terminal His tag for expression in *Escherichia coli* strain BL21(DE3). The affinity‐purified recombinant proteins were incubated with 5‐aminopental for 30 min and the product formed was analyzed using LC‐MS. Both enzymes exhibited aldehyde dehydrogenation activity towards 5‐aminopentanal, resulting in the formation of 5‐aminopentanoate (Figure S12). Thus, not only cadaverine but also its catabolites could be accepted as substrates by the Arabidopsis enzyme pool to drive the biosynthesis of specific metabolites in the transgenic lines.

### Isotope‐labeled l‐lysine was incorporated into metabolites specifically accumulated in DC lines

**Figure 4:**
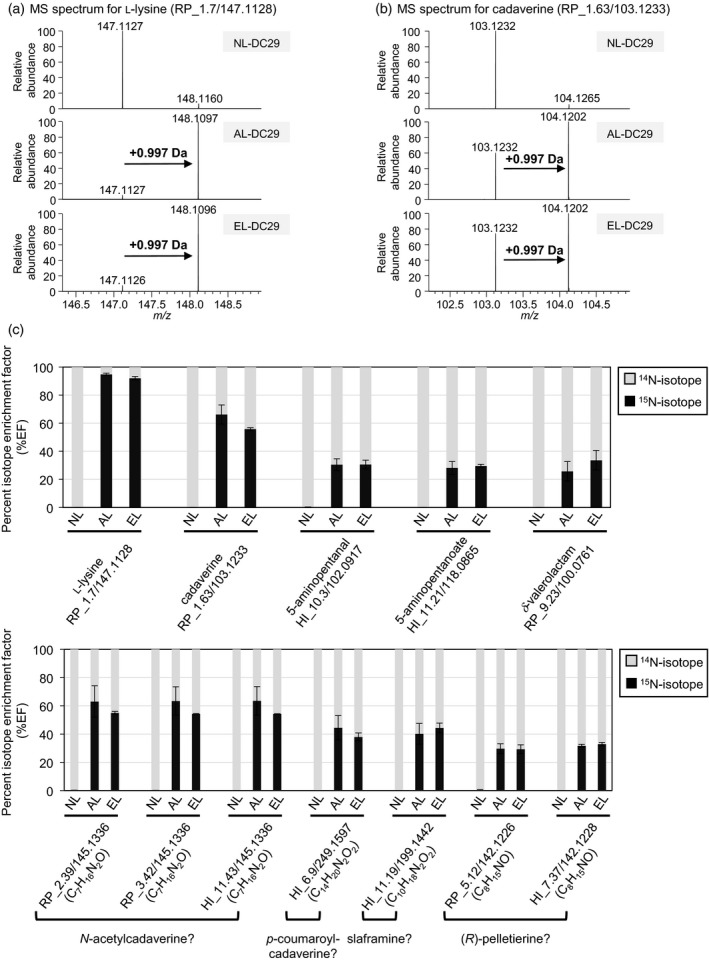
[α‐^15^N]‐ and [ε‐^15^N]‐l‐Lysine treatment of DC29 resulted in the incorporation of ^15^N into specific metabolite peaks. Isotopic mass shift in peaks of (a) l‐lysine (RP_1.7/147.1128) and (b) cadaverine (RP_1.63/103.1233) in plant extracts treated for 5 days with [α‐^15^N]‐l‐lysine (AL), [ε‐^15^N]‐l‐lysine (EL) or non‐labeled l‐lysine (NL). Mass shifts of 0.997 Da corresponding to the mass difference between ^15^N and ^14^N were observed in DC29 treated with AL or EL, whereas a natural abundance of the ^15^N isotopolog peak (around 0.7%) was detected in DC29 treated with NL. (c) Isotope enrichment factor (%EF) for specific metabolite peaks. Data represent means ± standard deviations (*n* = 3).

To further verify that specific metabolite features in DC lines were synthesized from l‐lysine via cadaverine, we fed the plants with ^15^N‐labeled l‐lysine and traced cadaverine catabolism in DC29. We used two types of stable isotope‐labeled l‐lysine, namely [α‐^15^N]‐ or [ε‐^15^N]‐l‐lysine (AL and EL, respectively). Taking into account the symmetric structure of cadaverine, we expected similar ratios of stable isotope labeling in cadaverine and cadaverine‐derived metabolites in plants fed with AL and EL. We fed 10‐day‐old seedlings of DC29 with AL, EL or non‐labeled l‐lysine (NL) and incubated them for 5 days before harvesting the seedlings for further analysis. LC‐MS analysis for the metabolites extracted showed a mass shift of 0.997 Da, corresponding to the mass difference between ^15^N and ^14^N, being observed for l‐lysine (RP_1.7/147.1128 and HI_25.59/147.1128) and cadaverine (RP1.63/103.1233 and HI_24/103.1234) in AL/EL‐treated plants, whereas a natural abundance of the ^15^N isotopolog peak (around 0.7%) was detected in DC29 treated with NL (Figure 4a,b). This result confirms that the exogenously applied l‐lysine was incorporated into the Arabidopsis metabolome and converted into cadaverine by La‐L/ODC.

Next, in order to evaluate the incorporation of exogenously applied l‐lysine into specific metabolite features, the percentage isotope enrichment factors (%EFs) were calculated using the following formula: %EF = [(intensity of M + 1)/(sum of intensities of M and M + 1)] × 100, where M and M + 1 represent the monoisotopic mass of an unlabeled metabolite and a labeled metabolite with a mass shift of 0.997 Da, respectively. The %EF values were further corrected by taking into account the natural abundance of ^15^N isotopolog peaks, as described previously (Campbell, 1974; Bunsupa *et al*., 2014). Around 92.8–95.4% of l‐lysine was labeled in DC29 treated with either AL or EL, whereas l‐lysine was not labeled in DC29 treated with NL (Figure 4c). Among all of the specific metabolite features, 17 and 24 metabolite features were labeled in both AL‐ and EL‐treated plants in RPLC and HILIC mode, respectively (Figure 4c; Tables 1 and S5). In the case of cadaverine, as expected the labeling ratios did not significantly differ between AL‐ and EL‐treated plants, being 66.7 and 56.2%, respectively (Figure 4c). As for 5‐aminopentanal (RP_2.34/102.0917 and HI_10.3/102.0917), 5‐aminopentanoate (HI_11.21/118.0865) and δ‐valerolactam (RP_9.23/100.0761), the labeling ratios were further reduced to around 25.9–34.1%, almost half the level of the labeling ratios found in cadaverine (Figure 4c; Table S5). These results indicate that one of two nitrogen atoms in cadaverine was lost during the synthesis of these metabolites. *N*‐Acetylcadaverine‐like peaks (RP_2.39/145.1336, RP_3.42/145.1336 and HI_11.43/145.1336) were labeled almost to the same level as that of cadaverine (54.7–64.1%), whereas the labeling ratio of the *p*‐coumaroylcadaverine‐like peak (HI_6.9/249.1597) was slightly lower than that of cadaverine (38.2–44.8%) (Figure 4c; Table S5). This implies that the rate of conversion of cadaverine to *p*‐coumaroylcadaverine is slower than the conversion rate to *N*‐acetylcadaverine. The slaframine‐like (HI_11.19/199.1442) and the (*R*)‐pelletierine‐like (RP_5.12_142.1226 and HI_7.37/142.1228) metabolite features were also equally labeled between AL‐ and EL‐treated plants (Figure 4c; Table S5), suggesting that these metabolites are also derived from cadaverine. All of these data clearly indicate that the exogenously applied l‐lysine pool was converted into symmetric cadaverine by the activity of La‐L/ODC, which in‐turn transformed into several specific metabolites.

## Discussion

The functional evolution of metabolic enzymes is the starting point of a new biosynthetic branch from primary metabolism, which may result in the production of several new metabolites (Khersonsky and Tawfik, 2010). It is widely speculated that some of these metabolites provide advantages to plants for survival under biotic/abiotic stress conditions (Schwab, 2003; Weng, 2014; Michael, 2017). In Lys‐derived alkaloid biosynthesis, the L/ODC enzyme serves at the branch point of primary metabolism and alkaloid biosynthesis. An amino acid substitution in L/ODC at position 344, which enhances LDC activity, has been reported to occur in distant plant species through positive convergent evolution (Bunsupa *et al*., 2016). This implies that new or increased cadaverine production in plants provides an advantage for survival under selective pressure. Although the detailed mechanism underlying the evolution of Lys‐derived alkaloid biosynthesis from cadaverine production remains unclear, this study revealed a possible role of cadaverine as an expander for metabolite diversity in Arabidopsis as a first step towards the emergence of alkaloid biosynthesis.

### Arabidopsis may cope with an increased accumulation of putrescine and cadaverine by acyl conjugation

The differential metabolome analysis for DC lines and Col‐0 revealed an upregulation of phenylpropanoid biosynthesis in DC lines (Figures 2e and S7). One of the key metabolite groups of phenylpropanoid biosynthesis is hydroxycinnamoyl‐CoAs, which provide acyl moieties for the conjugation of polyamines. In plants, the accumulation of putrescine over a specific threshold is lethal (DeScenzo and Minocha, 1993; Masgrau *et al*., 1997; Alcázar *et al*., 2005). Thus, Alcázar *et al*. (2005) reported that the increased putrescine levels enhanced the accumulation level of putrescine conjugates in Arabidopsis, which probably contributed to the decrease in the cellular concentration of free polyamine levels. Consistent with previous reports, DC lines showed a higher accumulation of putrescine and *p*‐coumaroylputrescine compared with Col‐0 (Figures S2b and S6; Table S4). DC lines also showed an increase in the expression of *agmatine coumaroyltransferase* (*AtACT*; At5 g61160), a gene encoding an enzyme responsible for the conjugation of putrescine with hydroxycinnamoyl‐CoAs (Figure S13) (Muroi *et al*., 2009). Therefore, these results suggest that Arabidopsis copes with increased putrescine and cadaverine levels by promoting acyl conjugation to prevent free polyamines from accumulating to toxic levels.

### The accumulation of γ‐l‐glutamylputrescine implies the existence of an unknown putrescine catabolic mechanism

Among differentially accumulated putrescine and putrescine‐derived metabolites in the DC lines (Figure S6), γ‐l‐glutamylputrescine has not yet been reported in plants but is known to be synthesized in prokaryotes by γ‐glutamylpolyamine synthetase (Krysenko *et al*., 2017). The homologs of this enzyme seem to be widespread across the plant kingdom. In Arabidopsis, At3g53180 represents the homolog of functionally characterized γ‐glutamylpolyamine synthase from *Streptomyces coelicolor* M145 (SCO6962). Although the molecular function of At3g53180 remains unclear, its mutant results in the inhibition of the main root growth, reduced meiotic activity in the cell division zone and disorder of root cap development (Doskočilová *et al*., 2011). Further functional characterization of At3g53180 may reveal a novel putrescine catabolic pathway in plants.

### Feeding with isotope‐labeled l‐lysine revealed cadaverine‐derived metabolites in DC lines

Feeding with AL, EL or NL resulted in 17 (RPLC mode) and 23 (HILIC mode) specific metabolite features being labeled in both AL‐ and EL‐treated DC29 plants (Tables 1; Table S5). Previously, feeding AL or EL to the hairy roots of *Nicotiana tabacum* harboring *La‐L/ODC* resulted in a similar labeling ratio of anabasine, an Lys‐derived alkaloid (Bunsupa *et al*., 2014), which confirmed that anabasine was derived from cadaverine. Our results also show similar ^15^N incorporation levels for the majority of specific metabolite features labeled in AL‐ and EL‐treated DC29 plants, suggesting that these metabolites are derived from cadaverine. Taking the chemical structure into account, the same labeling ratio as that of cadaverine for specific metabolite features would suggest both nitrogen atoms being derived from cadaverine. On the other hand, metabolites losing one nitrogen in the course of bioconversion from cadaverine would be labeled with almost half the level found in cadaverine. Consistent with this hypothesis, several specific metabolites with one nitrogen atom in their predicted chemical formula were labeled with almost half the level found in cadaverine (Table S5). The labeling ratios of 5‐aminopentanal, 5‐aminopentanoate and δ‐valerolactam were nearly half the level of that labeled in cadaverine, thus suggesting the loss of one nitrogen atom in the process of bioconversion from cadaverine. For *N*‐acetylcadaverine, the labeling ratio was almost at the same level as that of cadaverine (Figure 4c; Table S5), thus suggesting that this metabolite is synthesized through a simple *N*‐acetylation reaction of labeled cadaverine. Contrary to our hypothesis, *p*‐coumaroylcadaverine, *N*‐acylated cadaverine, was labeled at a level marginally lower than that of cadaverine (Figure 4c; Table S5), which could result from a slow conversion rate of cadaverine into *p*‐coumaroylcadaverine. The only exception where specific metabolite features were not labeled after being fed with both AL and EL was HI_11.19/178.0241, labeled only in the AL‐treated DC29 plants (Table S5). This metabolite might be transformed from l‐lysine by the regiospecific elimination of ε‐^15^N and then synthesized without passing through cadaverine. Further study will be required to elucidate this mechanism as the chemical structure for this metabolite could not be predicted by ms‐finder. Among specific peaks, several metabolite features were not labeled with isotope (29 peaks in RPLC mode and eight peaks in HILIC mode) (Table S5). These metabolites could lose both nitrogen atoms during synthesis or be non‐cadaverine‐derived metabolites accumulated in response to *La‐L/ODC* expression.

### Cadaverine catabolic pathway in Arabidopsis emerged by ectopic *La‐L/ODC* expression

**Figure 5:**
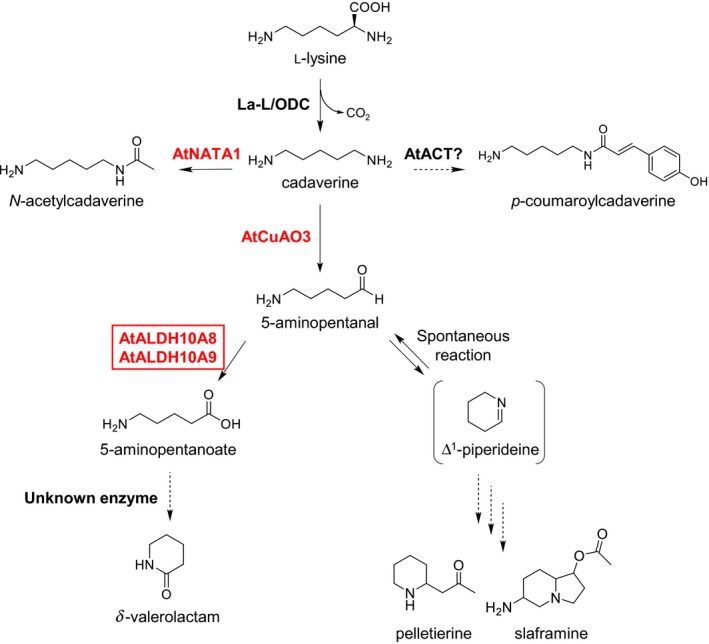
Proposed cadaverine catabolic pathway in Arabidopsis. By the action of exogenous La‐L/ODC, l‐lysine is converted into cadaverine, which further undergoes three major catabolic pathways: 1, oxidation leading to biosynthesis of δ‐valerolactam and alkaloid‐like metabolites; 2, *N*‐acetylation; and 3, conjugation with *p*‐coumaroyl‐CoA. In pathway 1, cadaverine is oxidized to 5‐aminopentanal, and then this metabolite is further converted into either 5‐aminopentanoate and δ‐valerolactam or is spontaneously transformed into Δ^1^‐piperideine to lead to compounds such as (*R*)‐pelletierine‐like and slaframine‐like metabolites. Pathways 2 and 3 form *N*‐acetylcadaverine and *p*‐coumaroylcadaverine, respectively. Enzymes in red have been shown to exhibit activity towards cadaverine or cadaverine catabolites. The activities of AtALDH10A8 and AtALDH10A9 against 5‐aminopentanal were confirmed in this study. Abbreviations: AtACT, agmatine coumaroyltransferase; AtALDH, aldehyde dehydrogenase; AtNATA1, *N*‐acetyltransferase activity1; AtAO1, amine oxidase1; La‐L/ODC, lysine/ornithine decarboxylase.

Among the cadaverine‐derived metabolites identified in the transgenic lines, 5‐aminopentanal, 5‐aminopentanoate, *N*‐acetylcadaverine and *p*‐coumaroylcadaverine are analogs of four of the putrescine‐derived metabolites, namely 4‐aminobutanal, γ‐aminobutyric acid (GABA), *N*‐acetylputrescine and *p*‐coumaroylputresine, respectively. Compared with cadaverine catabolism, the putrescine catabolic pathway is well characterized, including metabolite intermediates and the enzymes involved (Figure S14). Putrescine and cadaverine are analogs with a difference of one carbon atom. Previous studies have shown that several enzymes involved in putrescine catabolism could also accept cadaverine or cadaverine catabolites as substrates (Jammes *et al*., 2014; Naconsie *et al*., 2014; Zarei *et al*., 2015). Taking into account the putrescine catabolic pathway and considerations based on the principles of organic chemistry for the specific metabolites identified in this study, we propose the cadaverine catabolic pathway in Arabidopsis (Figure 5). Our results suggest three possible metabolic fates for cadaverine in Arabidopsis: (i) oxidation leading to the biosynthesis of δ‐valerolactam and alkaloid‐like metabolites; (ii) *N*‐acetylation; and (iii) conjugation with *p*‐coumaroyl‐CoA.

In the biosynthesis of δ‐valerolactam and alkaloid‐like metabolites, cadaverine first undergoes oxidation to form 5‐aminopentanal. Biosynthesis of 5‐aminopentanal from cadaverine is known to be catalyzed by CuAO. There are 10 genes annotated as CuAO or CuAO‐like enzymes, eight of which encode putative functional enzymes in Arabidopsis: AtAO1 (At4g14940), AtCuAO1 (At1g62810), AtCuAO2 (At1g31710), AtCuAO3 (At2g42490), AtCuAOα1 (At1g31670), AtCuAOα2 (At1g31690), AtCuAOγ2 (At3g43670) and AtCuAOδ (At4g12290) (Tavladoraki *et al*., 2016). Phylogenetic analysis using all eight CuAO or CuAO‐like enzymes from Arabidopsis with other plant CuAOs resulted in the formation of three major clades (Figure S15). Among the eight Arabidopsis CuAOs, AtCuAO3 was classified in clade III, which included four previously characterized enzymes from *L. angustifolius* (LaCuAO), *N. tabacum* (NtDAO1 and NtMPO) and *Malus domestica* (MdAO1), all of which have shown activity towards cadaverine (Naconsie *et al*., 2014; Zarei *et al*., 2015; Yang *et al*., 2017). Furthermore, LaCuAO oxidizes cadaverine to synthesize 5‐aminopentanal, an important biosynthetic intermediate of Lys‐derived alkaloid. Previously, AtAO1, AtCuAO1, AtCuAO2 and AtCuAO3 have been functionally characterized and shown to catalyze the oxidation of aliphatic amines, including putrescine (Moller and McPherson, 1998; Wimalasekera *et al*., 2011; Planas‐Portell *et al*., 2013; Tavladoraki *et al*., 2016). Among these, Naconsie *et al*. (2014) reported that AtCuAO3 also accepts cadaverine as a substrate, but with a lower catalytic efficiency compared with putrescine. Therefore, AtCuAO3 seems to be a potent candidate responsible for the oxidation of cadaverine in DC lines. Gene expression analysis showed a marginal upregulation of *AtAO1*,* AtCuAO1* and *AtCuAO3*, whereas *AtCuAO2* was slightly downregulated in DC29 with respect to Col‐0 (Figure S13).

5‐Aminopentanal, thus formed, is then transformed into 5‐aminopentanoate or spontaneously cyclized into Δ^1^‐piperideine. Arabidopsis does not produce 5‐aminopentanal or 5‐aminopentanoate but rather produces analogs that are one carbon atom shorter: 4‐aminobutanal and GABA, respectively. The conversion of 4‐aminobutanal to GABA was previously shown to be catalyzed by AtALDH10A8 and AtALDH10A9 (Missihoun *et al*., 2011; Stiti *et al*., 2011; Zarei *et al*., 2016). In this study, we functionally characterized AtALDH10A8 and AtALDH10A9 enzymes and showed that both enzymes could catalyze the conversion of 5‐aminopentanal to 5‐aminopentanoate. Thus, both AtALDH10A8 and AtALDH10A9 serve as co‐opting enzymes from the non‐cadaverine‐producing plant Arabidopsis to catabolize newly synthesized cadaverine metabolic intermediates. The expression levels of these two genes were also slightly increased in DC29 compared with Col‐0 (Figure S13).

5‐Aminopentanoate is further cyclized to form δ‐valerolactam; however, at this point, little is known about the enzymes in Arabidopsis that may catalyze this reaction. There has been no report on δ‐valerolactam identification or accumulation in plants, and the enzyme family responsible for the formation of this compound is unclear. In animals, crude lipase from pancreatic porcine exhibits lactamization activity to synthesize δ‐valerolactam (Gutman *et al*., 1992). Thus, a lipase‐like protein in Arabidopsis might catalyze this reaction.

5‐Aminopentanal is also spontaneously cyclized into Δ^1^‐piperideine, which has been demonstrated as the biosynthetic intermediate for several Lys‐derived alkaloids including quinolizidine, lycopodium and piperidine through isotope tracing (Braekman *et al*., 1972; Leeper *et al*., 1981; Golebiewski and Spenser, 1985) and computational chemistry‐based predictions (Sato *et al*., 2018). Interestingly, MS/MS‐based structure predictions using ms‐finder annotated several specific metabolite features as Lys‐derived alkaloids, including slaframine (HI_11.19/199.1442) and (*R*)‐pelletierine (RP_5.12/142.1226 and HI_7.37/142.1228), both of which were labeled by feeding with stable isotope‐labeled l‐lysine (Figure 4c; Table S5). Other than these two metabolites, the predicted candidates of several metabolite intermediates contained the piperidine structure, such as 3‐quinucidinol (RP2.39/128.1071), lentiginosine (HI_6.18/158.1177) and UNPD117128 (HI_6.74/181.1336) (Table S5). These data imply that Δ^1^‐piperideine, derived from cadaverine in DC lines, would serve as the intermediate for alkaloid‐like metabolites to increase the chemical diversity found in DC lines.

The second metabolic fate of cadaverine in the form of *N*‐acetylation could be catalyzed by AtNATA1, the expression of which was also moderately increased in the DC29 line (Figure S13). Previously, AtNATA1 has been shown to catalyze 1,3‐diaminopropane, an analog of cadaverine that is two carbon atoms shorter, and was also shown to possess activity against cadaverine (Jammes *et al*., 2014; Lou *et al*., 2016). As DC lines showed a high cellular content of cadaverine (0.04–1.25 nmol mg^−1^ FW) (Figure 1b), AtNATA1 could co‐opt cadaverine as a substrate resulting in the synthesis of *N*‐acetylcadaverine. The third metabolic fate of cadaverine, namely the conjugation of cadaverine with the *p*‐coumaroyl moiety, is most likely catalyzed by AtACT, the expression of which was also significantly increased in the DC lines (Figure S13). AtACT catalyzes putrescine conjugation with certain acyl donors (*p*‐coumaroyl‐CoA or feruloyl‐CoA) to produce phenolamides, namely *p*‐coumaroylputrescine and feruloylputrescine (Muroi *et al*., 2009). Based on the catalytic property of AtACT, we expected to identify both *p*‐coumaroylcadverine and feruloylcadaverine as specific peaks in DC lines. Although we detected *p*‐coumaroylcadverine as the specific metabolite feature, feruloylcadaverine was not detected in DC lines (Figure S6; Table S4). Considering that feruloylputrescine is a minor metabolite in Arabidopsis and that AtACT prefers *p*‐coumaroyl‐CoA over feruloyl‐CoA as a substrate (Muroi *et al*., 2009), it is likely that the intensity of the feruloylcadaverine peak was below the cut‐off value and was excluded as noise in our analysis. Taken together, our results show that Arabidopsis, a plant that does not produce cadaverine or its intermediates, has a pool of enzymes with  promiscuous activities that produce several downstream metabolites.

Previously, Bunsupa *et al*. (2016) reported convergent evolution from the ODC enzyme to the L/ODC enzyme in distant plant species, producing Lys‐derived alkaloids under positive selection. Similar observations were also made for other plant species producing benzylisoquinoline alkaloids (BIAs) and tropane alkaloids (Liscombe *et al*., 2005; Jirschitzka *et al*., 2012). These observations led to the hypothesis that plants originally possessed metabolic machinery for the onward catabolism of newly emerged metabolites in order to derive biosynthetic pathways for specialized metabolites. Thus, cadaverine production acquired from LDC activity seems to be co‐opted by enzymes involved in other pathways to synthesize various metabolites, which provides an evolutionary path for the expansion of alkaloid diversity.

### Co‐opting enzymes undergo gene expansion for the evolution of cadaverine‐derived metabolite biosynthesis

Gene expansion followed by neo/sub‐functionalization plays an important role in the diversification of secondary metabolites (Rai *et al*., 2017a, 2017b). Therefore, we evaluated the gene expansion of candidate genes assigned to emerged cadaverine catabolic pathways across nine plant species: three non‐cadaverine and non‐functional‐ODC plants (*A. thaliana*, *Brassica rapa*, and *Physcomitrella patens*); two non‐cadaverine and functional‐ODC plants (*Malus domestica* and *Papaver somniferum*); and four cadaverine and functional‐L/ODC plants (*Glycine max*,* L. angustifolius*,* Medicago truncatula* and *Nelumbo nucifera*). The predicted genes from all nine plant species were categorized as orthogroups (by gene family) using the orthofinder tool (Emms and Kelly, 2015). Probabilities for a gene family to have undergone gain, expansion, loss or contraction were analyzed based on the species tree and the copy number of the assigned genes by computing posterior probabilities for the family sizes at the internal nodes using the count package (Csuos, 2010). orthofinder analysis resulted in a total of 20 444 orthogroups across nine plant species. Among these, 1297 orthogroups were species specific whereas 6628 orthogroups contained at least one gene from each of the species. We focused on the orthogroups corresponding to the enzymes assigned to the cadaverine catabolic pathway in Arabidopsis (Figures 5 and S16; Table S6), such as L/ODC (OG0011669), AtCuAO3 (OG0001988), AtALDH10A8/AtALDH10A9 (OG0006295), AtACT (OG0000305) and AtNATA1 (OG0009526). Among these orthogroups, only OG0011669 (L/ODC or ODC) showed a significant gain at node 6 (Figure S16a; Table S6), whereas all other orthogroups showed no significant gain specific to any of the plant species nodes. Although the evolution of ODC has been attributed to specialized metabolite biosynthesis (Bunsupa *et al*., 2012), all other orthogroups are involved in the primary or essential metabolic processes, and thus no significant probability for gene gain except for OG0011669 (L/ODC) is expected. Regarding gene expansion, high probabilities for orthogroups corresponding to *L/ODC*,* AtALDH10A8*,* AtALDH10A9* and *AtNATA1* were observed in particular for *L. angustifolius*,* Malus domestica* and *Papaver somniferum* (Figure S16; Table S6). Furthermore, *Medicago truncatula* and *G. max* also showed high probabilities for gene expansion for *L/ODC* (Figure S16; Table S6). OG0001988, which corresponds to CuAOs from clade III, contained a single gene copy from Arabidopsis but multiple gene copies from other plant species, including five genes from *L. angustifolius* contained in this orthogroup (Figure S16; Table S6). In terms of gene expansion for OG0001988, only *B. rapa* showed high probability (as the copy number increased from one to two copies; Figure S16; Table S6). Nevertheless, increased copy number for genes corresponding to *AtCuAO3* in these plant species suggests an evolutionary role of CuAOs from clade III in the specialized metabolite biosynthesis. Our results, therefore, imply that the enzymes that play an important role towards primary or essential metabolism across different plant species may co‐opt cadaverine to produce specialized metabolites, and could be the basis for the emergence of alkaloid biosynthetic pathways. Taken together, the cadaverine catabolic pathway elucidated in this study has potential implications for understanding how cadaverine in plants has expanded plant chemodiversity through the activities of endogenous enzymes.

## Experimental Procedures

### Chemicals and reagents

l‐Lysine and 5‐aminopentanoate were purchased from Tokyo Chemical Industry Co., Ltd. (https://www.tcichemicals.com); cadaverine, δ‐valerolactam, 1‐formylpyrrolidine, 1,9‐diaminononane and β‐nicotinamide adenine dinucleotide (NAD^+^) were obtained from Sigma‐Aldrich (http://www.sigmaaldrich.com); 5‐aminopentanal was acquired from Activate Scientific (https://shop.activate-scientific.com); [α‐^15^N]‐l‐Lysine and [ε‐^15^N]‐l‐lysine were purchased from Cambridge Isotope Laboratories Inc. (http://www.isotope.com); l‐ornithine, putrescine and all LC‐MS‐grade buffers used for LC‐MS were purchased from FUJIFILM Wako Pure Chemical Co. (http://www.wako-chem.co.jp).

### Vector construction, transformation and plant growth conditions

The full‐length coding sequence of *La*‐*L/ODC* was transferred to the pGWB502Ω vector (Nakagawa *et al*., 2007) via Gateway technology (ThermoFisher Scientific, https://www.thermofisher.com). Subsequently, the constructed vector was introduced into *Agrobacterium tumefaciens* strain GV3101 by the electroporation method and transformed into Col‐0 as described previously (Kim *et al*., 2012). Transgenic seedlings were selected by hygromycin (50 μg ml^−1^) on Murashige and Skoog agar plates, and 10 independent T_3_ generation lines were established.

Seeds were surface sterilized by a solution of 1% sodium hypochlorite with 0.1% Triton X‐100 for 10 min, followed by five washes with autoclaved water and were transferred to half‐strength Murashige and Skoog medium containing 1.5% sucrose and 0.8% agar (pH 5.8). After treatment at 4°C for 2 days, seeds were incubated at 22°C in a plant growth chamber with a 16‐h light/8‐h dark cycle. Two‐week‐old seedlings (30 plants) from each plate were harvested (regarded as one biological replicate), frozen in liquid nitrogen and stored at −70°C until use. For root‐length measurement, Murashige and Skoog agar plates were placed at an angle of 85° and maintained for 2 weeks under the aforementioned conditions.

### Gene expression analysis

Total RNA was prepared from 2‐week‐old seedlings using the RNeasy plant mini kit (QIAGEN, https://www.qiagen.com) as directed by the manufacturer. The total RNA sample (2 μg) for each biological replicate was used to derive single‐stranded cDNA using the SuperScript VILO cDNA Synthesis Kit (ThermoFisher Scientific), as directed by the manufacturer. The primer sequences used in this study are provided in Table S7. For semi‐quantitative RT‐PCR analysis, PCR was performed using LaL/ODC‐F and LaL/ODC‐R for *La‐L/ODC*, and using Tub‐sq‐F and Tub‐sq‐R for β*‐tubulin*. Semi‐quantitative RT‐PCR conditions were as follows: for *La‐L/ODC*, 28 cycles at 94°C for 30 s, 55°C for 30°s, and 72°C for 20 s; for β*‐tubulin*, 31 cycles at 94°C for 30 s, 55°C for 30 s, and 72°C for 40 s. PCR amplicons were analyzed by electrophoresis using 1.5% agarose gel stained by ethidium bromide and observed under UV light. Quantitative RT‐PCR was performed using Power SYBR® Green PCR Master Mix (ThermoFisher Scientific) on an Applied Biosystems StepOnePlus^™^ Real‐Time PCR System (ThermoFisher Scientific). The comparative cycle threshold method (ΔΔ*C*_T_) was used to calculate relative gene expression. All analyses were performed with four or five biological replicates.

### UHPLC‐MS‐based non‐targeted metabolome analysis

Metabolites were extracted as described previously (Nakabayashi *et al*., 2014). The metabolite extracts for three biological replicates of DC lines (DC21, DC29 and DC42) and Col‐0 were analyzed on a Dionex Ultimate 3000 RSLC HPLC (ThermoFisher Scientific) connected to an Orbitrap Q Exactive mass spectrometer (ThermoFisher Scientific). Metabolite extracts were separated by an InertSustain AQ‐C18 (3 μm, 2.1 mm × 150 mm, GL Sciences, https://www.glsciences.com) column (RPLC mode) or Inertsil Amide (3 μm, 2.1 mm × 150 mm, GL Sciences) column (HILIC mode), respectively. The column temperature was set at 40°C in both modes. For RPLC‐mode analysis, water (solvent A) and acetonitrile (solvent B) were acidified by 0.1% formic acid and used as the mobile phases. The gradient was set as follows: 0.0–3.0 min, 2% B; 3.0–30.0 min, 2–98% B; 30.0–35.0 min, 98.0% B; 35.0–35.1 min, 98–2% B; 35.1–40.0 min, 2% B, at a flow rate of 0.3 ml min^–1^. For HILIC‐mode analysis, 10 mm ammonium formate (pH 2.5) (solvent A) and acetonitrile (solvent B) were used as the mobile phases. The gradient for the HILIC mode was set as follows: 0.0–30.0 min, 90–60% B; 30.0–35.0 min, 60% B; 35.0–35.1 min, 60–90% B; 35.1–50 min, 90% B, at a flow rate of 0.3 ml min^−1^. Ion source conditions were as follows: spray voltage, 3.2 kV; capillary temperature, 300°C; probe heat seal temperature, 400°C; sheath gas, 45 arbitrary units; aux gas, 10 arbitrary units; S‐lens RF level, 55. The MS acquisition setting was one full MS scan (*m/z* 50.0–750.0) followed by 10 data‐dependent MS/MS scans. The resolution was set to 70 000 and 17 500 for the full MS scan and MS/MS scans, respectively. MS/MS fragments were obtained by higher energy collision dissociation (HCD) with stepped normalized collision set at 30, 60 and 90% to enhance MS fragmentation, and the isolation width was *m/z* 2. Raw mass signals were acquired with xcalibur 4.1 (ThermoFisher Scientific).

For metabolite quantification by targeted metabolite analysis, 200 μl of 80:20 MeOH/H_2_O (v/v) solution containing 1 μg ml^−1^ of 1‐formylpyrrolidine and 1,9‐diaminononane (internal standards for RPLC and HILIC modes, respectively) was added per 100 mg of frozen sample. The remaining metabolite extraction method was the same as described above. Extracted metabolites were analyzed by the Agilent 1260 Infinity II HPLC system (Agilent Technologies, https://www.chem-agilent.com) in line with an Agilent 6120 mass detector equipped with an electrospray ion source in positive‐ion mode, as described previously (Rai *et al*., 2018). Injection volumes for metabolite extracts were 10 μl for RPLC mode and 5 μl for HILIC‐mode, respectively. The LC‐MS solvents were the same as those used for UHPLC‐MS‐based metabolome analyses. In RPLC mode, metabolites were separated by a Mightysil RP‐18 column (GP250‐4.6, 5 μm; Kanto Chemical, https://www.kanto.co.jp>). The gradient was set as follows: 0.0–5.0 min, 20% B; 5.0–10.0 min, 20–100% B; 10.0–17.0 min, 100.0% B; 17.0–17.1 min, 100–20% B; 17.1–22.0 min, 20% B, at a flow rate of 0.8 ml min^−1^. For the HILIC mode, the same column for non‐targeted metabolome analysis was used. The gradient was set as follows: 0.0–30.0 min, 90–55% B; 30.0–40.0 min, 55% B; 40.0–40.1 min, 55–90% B; 40.1–60.0 min, 90% B, at a flow rate of 0.3 ml min^−1^. The monitoring ions for single‐ion monitoring (SIM) mode consisted of a parent ion ([M+H]^+^) and a daughter ion with the highest intensity for each metabolite, which was selected based on the MS of authentic standards. δ‐Valerolactam and 1‐formylpyrrolidine were analyzed in RPLC mode, whereas the other metabolites were quantified in HILIC mode. The mass values used for SIM were as follows: l‐lysine, parent ion = 147.1, daughter ion = 130.1; cadaverine, parent ion = 103.1, daughter ion = 86.1; 5‐aminopentanal, parent ion = 102.1, daughter ion = 84.1; 5‐aminopentanoate, parent ion = 118.1, daughter ion = 101.1; δ‐valerolactam, parent ion = 100.1 (only the parent ion was monitored because the abundance of the daughter ion was quite low, <1%); l‐ornithine, parent ion = 133.1, daughter ion = 116.1; putrescine, parent ion = 89.1, daughter ion = 72.1; GABA, parent ion = 104.1, daughter ion = 87.1; 1‐formylpyrrolidine (internal standard for RPLC mode), parent ion = 100.1; 1,9‐diaminononane (internal standard for HILIC mode), parent ion = 159.2. The fragmentor voltage was set at 70 V and the other MS settings were the same as described previously (Rai *et al*., 2018). Dose–response curves for each metabolite were created based on the peak areas for standard compounds at different concentrations, and these were used to determine metabolite accumulation levels in the DC lines and in Col‐0. Microsoft excel software was used to calculate the metabolite contents from the calibration curves.

### Metabolome data processing and pathway enrichment analysis

The non‐targeted LC‐MS data files were converted into .mzXML files using msconvert (proteowizard 3.0, http://proteowizard.sourceforge.net) (Chambers *et al*., 2012), followed by feature extraction and peak alignment using powerget 3.5.8 ( http://www.kazusa.or.jp/komics/software/PowerGet) (Sakurai *et al*., 2014), as described previously (Kera *et al*., 2018), with intensity cut‐off values set at 8000 for RPLC mode and 3000 for HILIC mode, respectively. The data matrix thus obtained was used for PCA and OPLS‐DA by simca 13.0.3 (Umetrics, https://umetrics.com). We used an S‐plot, which combines the contribution/covariance (p[1]) and reliability/correlation (p(corr)[1]) by OPLS‐DA model, to select differential metabolite features associated with DC lines or Col‐0 (|p[1]| > 0.02 and |p(corr)[1]| > 0.02 were used as cut‐off values). These differential metabolite features were then annotated (with a mass tolerance of <10 ppm) with the KEGG Arabidopsis database (2018 version; Kanehisa and Goto, 2000; Kanehisa *et al*., 2014). Annotated metabolites were then mapped onto the KEGG metabolite pathway by kegarray 1.2.4 ( http://www.genome.jp/kegg) (Kotera *et al*., 2012), and *P* values for each metabolite pathway were calculated by Fisher's exact test. Metabolite peaks specifically detected in DC lines were obtained using the powermatch module in powerget by removing peaks detected in Col‐0 from the peak matrix. False‐positive mass features in the resulting peak matrix were further removed through manual validation by checking raw LC‐MS data.

### MS/MS‐based structure annotation

Both MS1 and MS/MS data for specific metabolite peaks were analyzed using ms‐finder 2.42 (Tsugawa *et al*., 2016). Default parameter settings were used except for mass tolerances (set at 10 ppm and 50 ppm for MS1 and MS/MS spectra, respectively), and metabolic *in silico* network expansions (MINEs) and the PubChem online databases were used only when there was no query in the local databases. The top three predicted compounds with a total score over five were selected as potential candidate metabolites for further analysis.

### Feeding experiments with stable isotope‐labeled l‐lysine

Ten surface‐sterilized seeds were transferred into 20 ml of half‐strength Murashige and Skoog medium containing 1.5% sucrose and grown at 22°C with a 16‐h light/8‐h dark cycle, with aeration by a rotary shaker NR‐3 (TAITEC, https://taitec.net) set at 100 rpm. A 200‐μl volume of 50 mm non‐labeled l‐lysine (NL), 50 mm [α‐^15^N]‐l‐lysine (AL) or 50 mm [ε‐^15^N]‐l‐lysine (EL) was added to the medium 10 days after incubation and seedlings were grown for a further 5 days. The pool of 10 seedlings from one flask was regarded as one biological replicate and three biological replicates for each treatment were used for metabolite profiling. Metabolite extraction, metabolome analysis, feature extraction and peak alignment were conducted as described above. Labeled features in AL‐ and EL‐treated plants were obtained using shiftedionsfinder ( http://www.kazusa.or.jp/komics/software/ShiftedIonsFinder) (Kera *et al*., 2014), as described previously (Kera *et al*., 2018), with the following parameters: Max fold, N  =  1; Mass difference  = 5 ppm; and RT difference  = 0.1. The ^15^N‐labeled features thus obtained were further curated manually. The percentage isotope enrichment factors (%EF) were calculated using the following formula: %EF = [(intensity of M + 1)/(sum of intensities of M and M + 1)] × 100, where M and M + 1 represents a monoisotopic mass of an unlabeled metabolite and a labeled metabolite with a mass shift of 0.997 Da, respectively. The %EF values were further corrected using the peak areas of ^15^N isotopolog peaks in NL, as described previously (Campbell, 1974; Bunsupa *et al*., 2014).

### Phylogenetic analysis

Amino acid sequences were aligned with clustalw and the phylogenetic tree was generated using the neighbor‐joining method in mega 7. The bootstrap values obtained with 1000 replicates are shown next to the branches. The evolutionary distances were computed using the Poisson correction method.

### Functional assay using recombinant AtALDH10A8 and AtALDH10A9

The full‐length cDNA clones of *AtALDH10A8* and *AtALDH10A9* were obtained from RIKEN BioResource Research Center ( https://ja.brc.riken.jp) (pda07810 and pda01165, respectively). Vector construction and the expression of recombinant protein followed by affinity purification were conducted as described previously (Zarei *et al*., 2016). The reaction mixture for the AtALDH10A8 functional assay consisted of 30 mm Tris‐HCl buffer (pH 8.5), 0.1 mm NAD^+^ and 1 mm 5‐aminopentanal, whereas the reaction mixture for AtALDH10A9 functional characterization contained 30 mm *N*‐cyclohexyl‐2‐aminoethanesulfonic acid buffer (pH 9.5), 0.5 mm NAD^+^ and 1 mm 5‐aminopentanal. Reactions were initiated by the addition of 5 μm recombinant protein in a total volume of 100 μl and incubated for 30 min at 25°C. The reactions were terminated by the addition of 900 μl of acetonitrile containing 1 μm 1,9‐diaminononane (internal standard), filtered through a 0.22‐μm filter (Merck Millipore, https://www.merckmillipore.com) and analyzed with LC‐MS under HILIC mode as described above.

### Gene expansion analysis

Protein sequences of nine plant species were downloaded from the the NCBI genome database. Accession IDs for genome assemblies of each plant are provided in Table S8. Orthologs of candidate genes were obtained using orthofinder 2.31, as described previously (Rai *et al*., 2017a, 2017b; Sun *et al*., 2018). Gene gain, loss, expansion and contraction in each orthogroup were evaluated in the count package, as noted previously (Li *et al*., 2018).

## Data Statement

Non‐targeted metabolome data are available in the supporting information. Raw data for other experiments are available upon request, from the corresponding author (mamiy@faculty.chiba-u.jp).

## Funding

This work was supported by the JSPS KAKENHI program (grant number 15H02494 to K.S. and 16H06454 to M.Y.), by the Strategic International Collaborative Research Program of Japan Science and Technology Agency (Metabolomics for a Low Carbon Society, JST‐NSF) and by the Strategic Priority Research Promotion Program of Chiba University.

## Author Contributions

KS and MY designed the research. YS, YO, HT and KK performed the experiments. YS, MS and HS acquired the metabolome data. YS and AR analyzed the data. YS, AR, KS and MY wrote the article.

## Conflict of Interest

The authors declare no conflicts of interest.

## Supporting information


**Figure S1.** Structure of binary vector and semi‐quantitative reverse transcription PCR analysis of DC lines.

**Figure S2.** Accumulation levels of l‐ornithine and putrescine in 2‐week‐old seedlings.

**Figure S3.** Root length and biomass of DC lines.

**Figure S4.** Experimental workflow.

**Figure S5.** R2 and Q2 values for the OPLS‐DA model.

**Figure S6.** Differential mass features associated with DC lines mapped to arginine and proline metabolism.

**Figure S7.** Differential mass features associated with DC lines mapped to phenylpropanoid biosynthesis.

**Figure S8.** Differential mass features in DC lines mapped to tropine, piperidine and pyridine alkaloid biosynthesis.

**Figure S9.** Differential mass features associated with DC lines mapped to biosynthesis of alkaloid derived from ornithine, lysine and nicotinic acid.

**Figure S10.** Differential mass features associated with Col‐0 mapped to the lysine degradation pathway.

**Figure S11.** Identification of cadaverine in DC lines.

**Figure S12.** Enzymatic conversion of 5‐aminopentanal to 5‐aminopentanoate by AtALDH10A8 and AtALDH10A9.

**Figure S13.** Expression analysis for candidate genes associated with cadaverine catabolism.

**Figure S14.** Putrescine metabolism in Arabidopsis.

**Figure S15.** Phylogenetic relationship of plant amine oxidases.

**Figure S16.** Gene gain, loss, expansion and contraction of candidate genes coding enzymes associated with cadaverine catabolism across nine plant species.

**Table S1.** All detected peaks.

**Table S2.** Results of OPLS‐DA.

**Table S3.** Differential mass features in DC lines and Col‐0.

**Table S4.** KEGG compound annotation for differential mass features.

**Table S5.** Annotation and labeling ratios for specific peaks in DC lines.

**Table S6.** Probability of gene gain, loss, expansion and contraction of candidate genes across nine plant species.

**Table S7.** Primers used in this study.

**Table S8.** Accession IDs of NCBI genomes used in this study.
