# Supplementary material for: Metabolic diversification of nitrogen‐containing metabolites by the expression of a heterologous lysine decarboxylase gene in Arabidopsis
Source: Plant J. 2019 Aug 27;100(3):505–21. doi: 10.1111/tpj.14454 (PMC6899585; doi:10.1111/tpj.14454)
Supplement: Supplementary file 1 — Figure S1. Structure of binary vector and semi‐quantitative reverse transcription PCR analysis of DC lines. [file TPJ-100-505-s001.pdf]

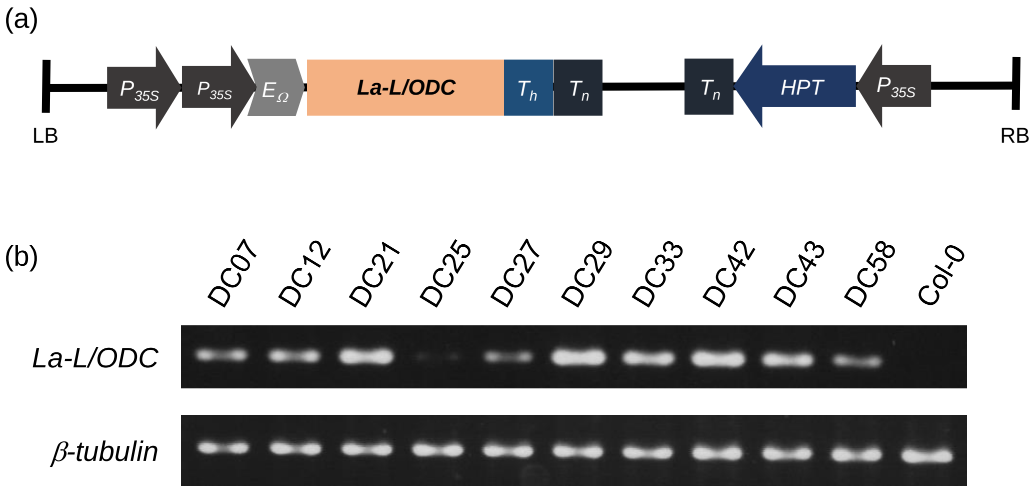

Figure S1. Structure of binary vector and semi-quantitative reverse transcription-PCR analysis of DC lines

(a) Schematic diagram of the T-DNA region of the binary vector used to generate transgenic plants. (b) Gel images of semi-quantitative RT-PCR products of *La-L/ODC* and *β-tubulin* in 10-independent T_3 transgenic lines. Thirty seedlings grown for two weeks were pooled and used for extraction of mRNA. Arabidopsis *β-tubulin* gene was used as an internal control. Based on band intensities, DC29, DC21 and DC42 were selected for further analysis. LB, left border; RB, right border; P_{35S}, *cauliflower mosaic virus 35S promoter*; E_{Ω}, *Ω enhancer*; *La-L/ODC*, *Lupinus angustifolius-lysine/ornithine decarboxylase*; *Th*, *heat shock protein terminator*; *Tn*, *nopaline synthase terminator*; *HPT*, *hygromycin phosphotransferase*.
