# Supplementary material for: Metabolic diversification of nitrogen‐containing metabolites by the expression of a heterologous lysine decarboxylase gene in Arabidopsis
Source: Plant J. 2019 Aug 27;100(3):505–21. doi: 10.1111/tpj.14454 (PMC6899585; doi:10.1111/tpj.14454)
Supplement: Supplementary file 2 — Figure S2. Accumulation levels of l‐ornithine and putrescine in 2‐week‐old seedlings. [file TPJ-100-505-s002.pdf]

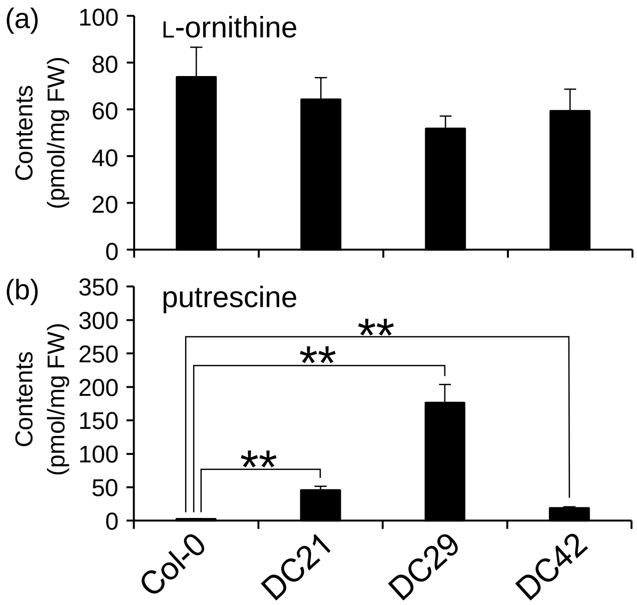

Figure S2. Accumulation levels of L-ornithine and putrescine in two-week-old seedlings
(a) L-Ornithine and (b) putrescine levels in metabolites extracted from two-week-old seedlings (30 seedlings were pooled and treated as one biological replicate) were quantified with LC-MS. Data are the mean ± standard error (*n* = 4-6). ***P* < 0.01 (Student's t-test). FW, fresh weight.
