# Supplementary material for: Metabolic diversification of nitrogen‐containing metabolites by the expression of a heterologous lysine decarboxylase gene in Arabidopsis
Source: Plant J. 2019 Aug 27;100(3):505–21. doi: 10.1111/tpj.14454 (PMC6899585; doi:10.1111/tpj.14454)
Supplement: Supplementary file 3 — Figure S3. Root length and biomass of DC lines. [file TPJ-100-505-s003.pdf]

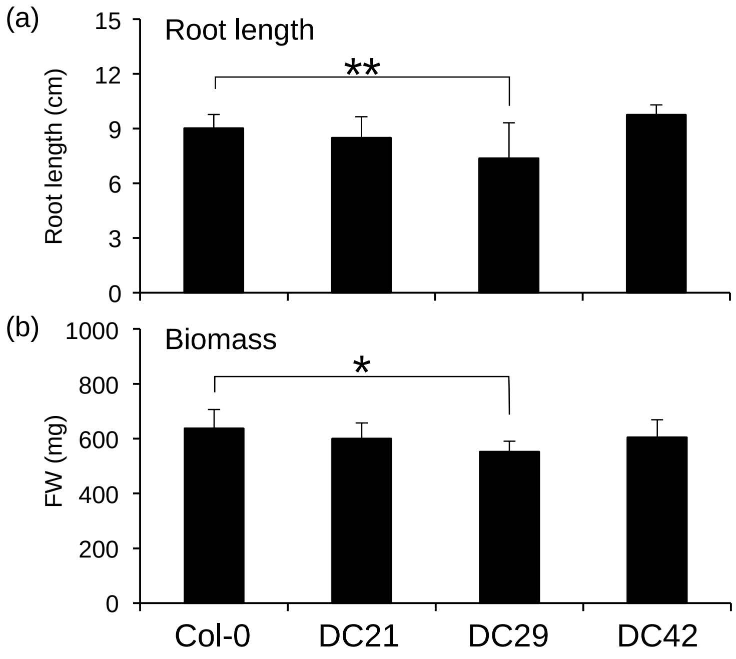

Figure S3. Root length and biomass of DC lines
(a) Comparison of root length of two-week-old DC lines. Values are means ± standard deviation (*n* = 6). (b) Biomass of seedlings grown for two weeks. Pooled two-week-old seedlings originating from 30 plants were regarded as one biological replicate for each line. Values are means ± standard deviation (*n* = 5). *$P < 0.05$, **$P < 0.01$ (Student's t-test). FW, fresh weight.
