# Supplementary figures and images for: Metabolic diversification of nitrogen‐containing metabolites by the expression of a heterologous lysine decarboxylase gene in Arabidopsis
Source: Plant J. 2019 Aug 27;100(3):505–21. doi: 10.1111/tpj.14454 (PMC6899585; doi:10.1111/tpj.14454)

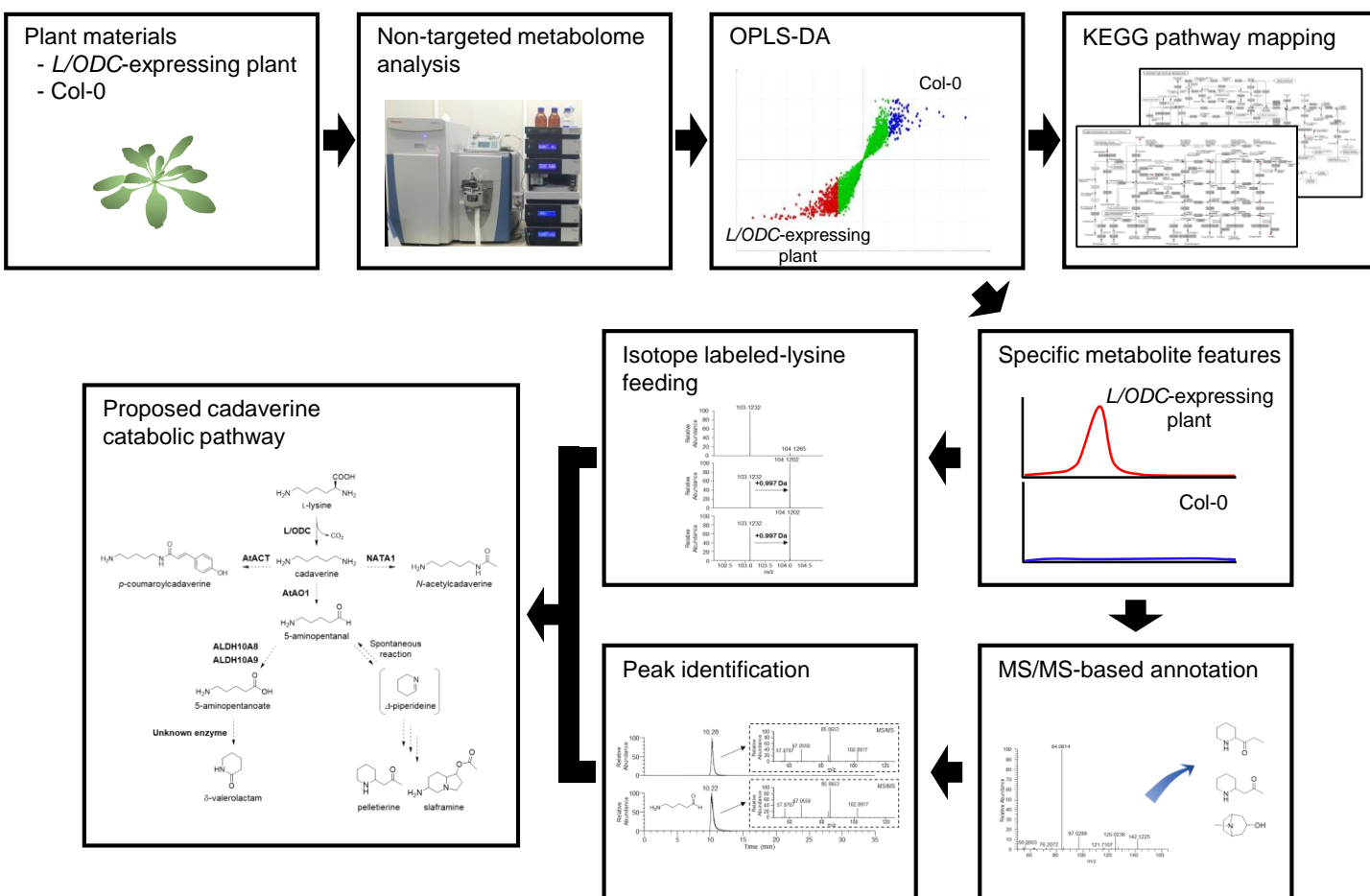

**Figure S4. Experimental workflow**

Supplement: Supplementary file 4 — Figure S4. Experimental workflow. [file TPJ-100-505-s004.pdf]

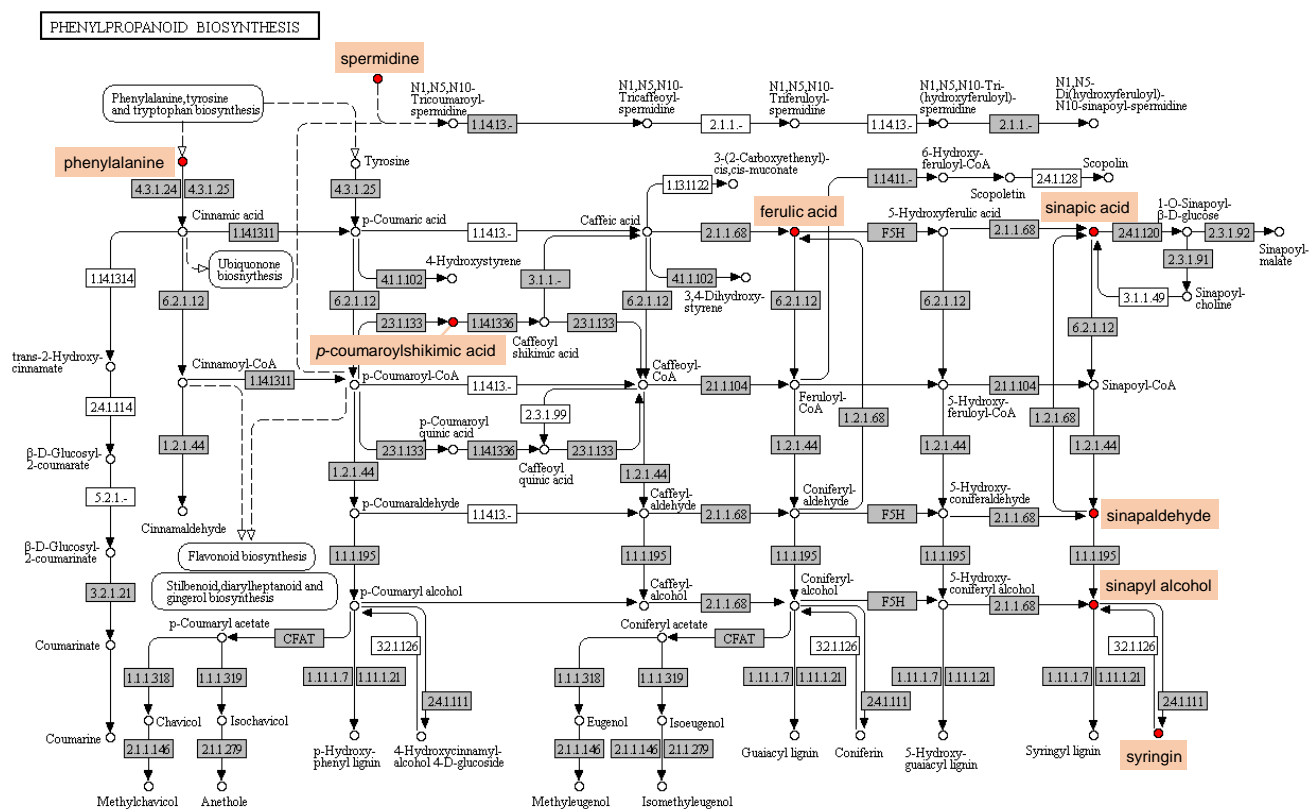

Supplement: Supplementary file 7 — Figure S7. Differential mass features associated with DC lines mapped to phenylpropanoid biosynthesis. [file TPJ-100-505-s007.pdf]
