# Supplementary material for: Metabolic diversification of nitrogen‐containing metabolites by the expression of a heterologous lysine decarboxylase gene in Arabidopsis
Source: Plant J. 2019 Aug 27;100(3):505–21. doi: 10.1111/tpj.14454 (PMC6899585; doi:10.1111/tpj.14454)
Supplement: Supplementary file 5 — Figure S5. R2 and Q2 values for the OPLS‐DA model. [file TPJ-100-505-s005.pdf]

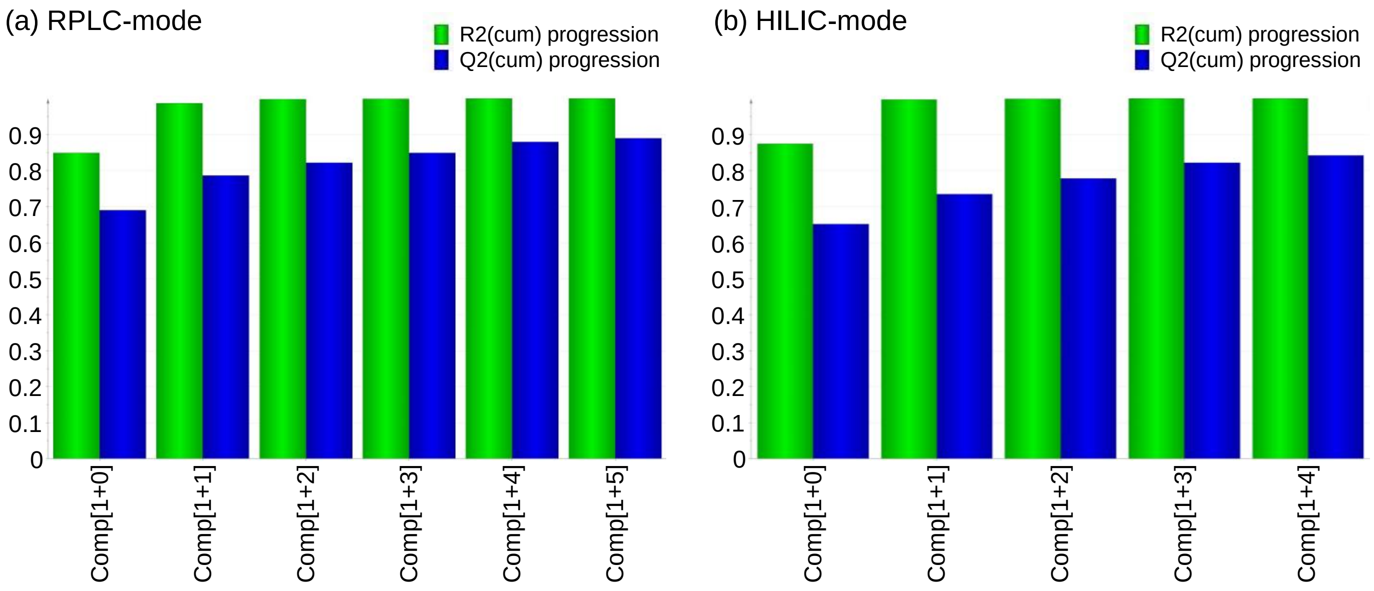

Figure S5. R2 and Q2 values for OPLS-DA model

Bar plots showing the R2 cumulative (green bar) representing the estimated fraction of total variance explained by the model, whereas Q2 cumulative (blue bar) indicates the accuracy of the model for prediction of variances. (a) RPLC-mode and (b) HILIC-mode.
