# Supplementary material for: Metabolic diversification of nitrogen‐containing metabolites by the expression of a heterologous lysine decarboxylase gene in Arabidopsis
Source: Plant J. 2019 Aug 27;100(3):505–21. doi: 10.1111/tpj.14454 (PMC6899585; doi:10.1111/tpj.14454)
Supplement: Supplementary file 6 — Figure S6. Differential mass features associated with DC lines mapped to arginine and proline metabolism. [file TPJ-100-505-s006.pdf]

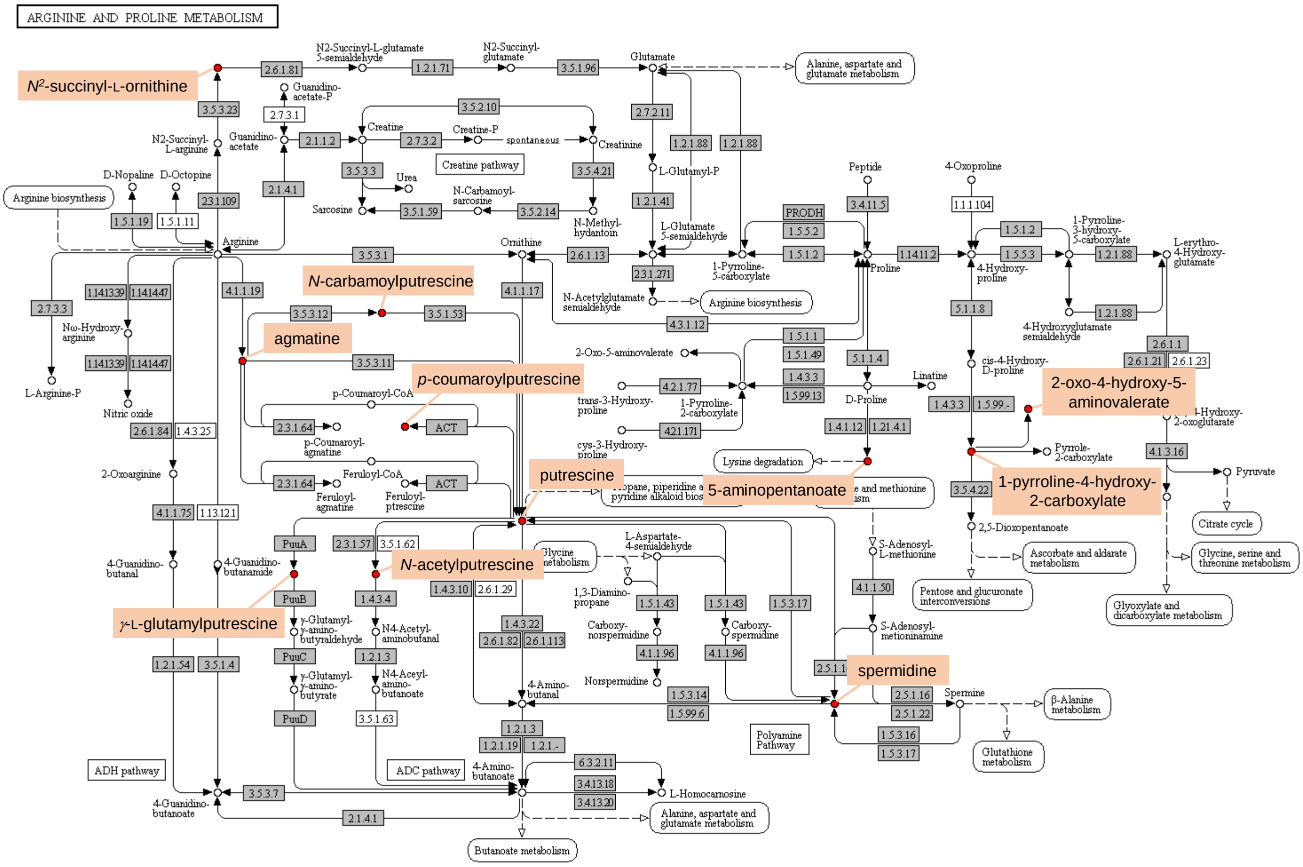

Figure S6. Differential mass features associated with DC lines mapped to arginine and proline metabolism

Differential metabolite features mapped to arginine and proline metabolism are shown in red, including putrescine, *p*-coumaroylputrescine, *N*-acetylputrescine, γ-L-glutamylputrescine, spermidine, N^2-succinyl-L-ornithine, *N*-carbamoylputrescine, agmatine, 5-aminopentanoate, 2-oxo-4-hydroxy-5-aminovalerate and 1-pyrroline-4-hydroxy-2-carboxylate.
