# Supplementary material for: Metabolic diversification of nitrogen‐containing metabolites by the expression of a heterologous lysine decarboxylase gene in Arabidopsis
Source: Plant J. 2019 Aug 27;100(3):505–21. doi: 10.1111/tpj.14454 (PMC6899585; doi:10.1111/tpj.14454)
Supplement: Supplementary file 8 — Figure S8. Differential mass features in DC lines mapped to tropine, piperidine and pyridine alkaloid biosynthesis. [file TPJ-100-505-s008.pdf]

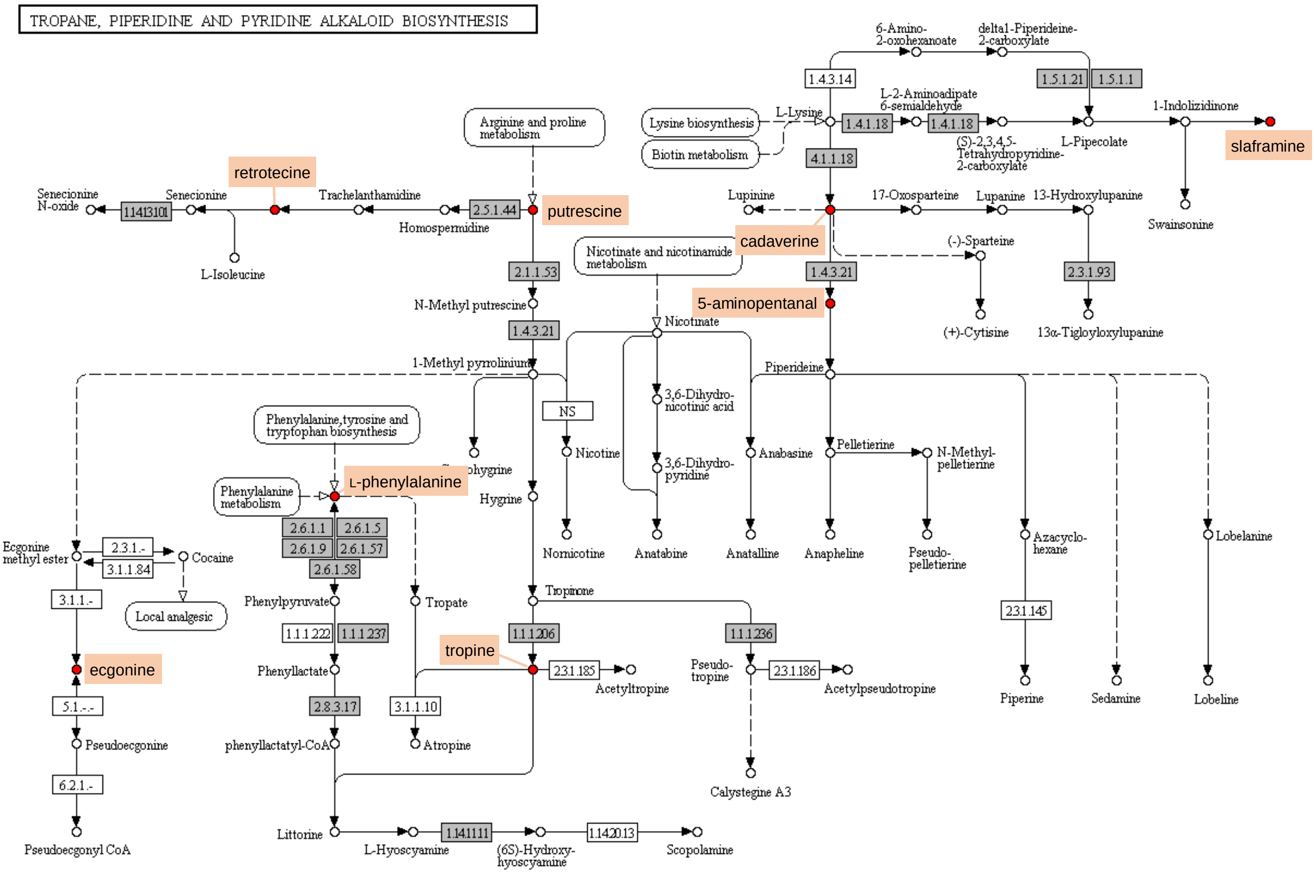

Figure S8. Differential mass features in DC lines mapped to tropine, piperidine and pyridine alkaloid biosynthesis

Differential mass features mapped to tropine, piperidine and pyridine alkaloid biosynthesis are shown in red, including cadaverine, 5-aminopentanal, slaframine, putrescine, retrotecine, tropine, L-phenylalanine and ecgonine.
