# Supplementary material for: Metabolic diversification of nitrogen‐containing metabolites by the expression of a heterologous lysine decarboxylase gene in Arabidopsis
Source: Plant J. 2019 Aug 27;100(3):505–21. doi: 10.1111/tpj.14454 (PMC6899585; doi:10.1111/tpj.14454)
Supplement: Supplementary file 9 — Figure S9. Differential mass features associated with DC lines mapped to biosynthesis of alkaloid derived from ornithine, lysine and nicotinic acid. [file TPJ-100-505-s009.pdf]

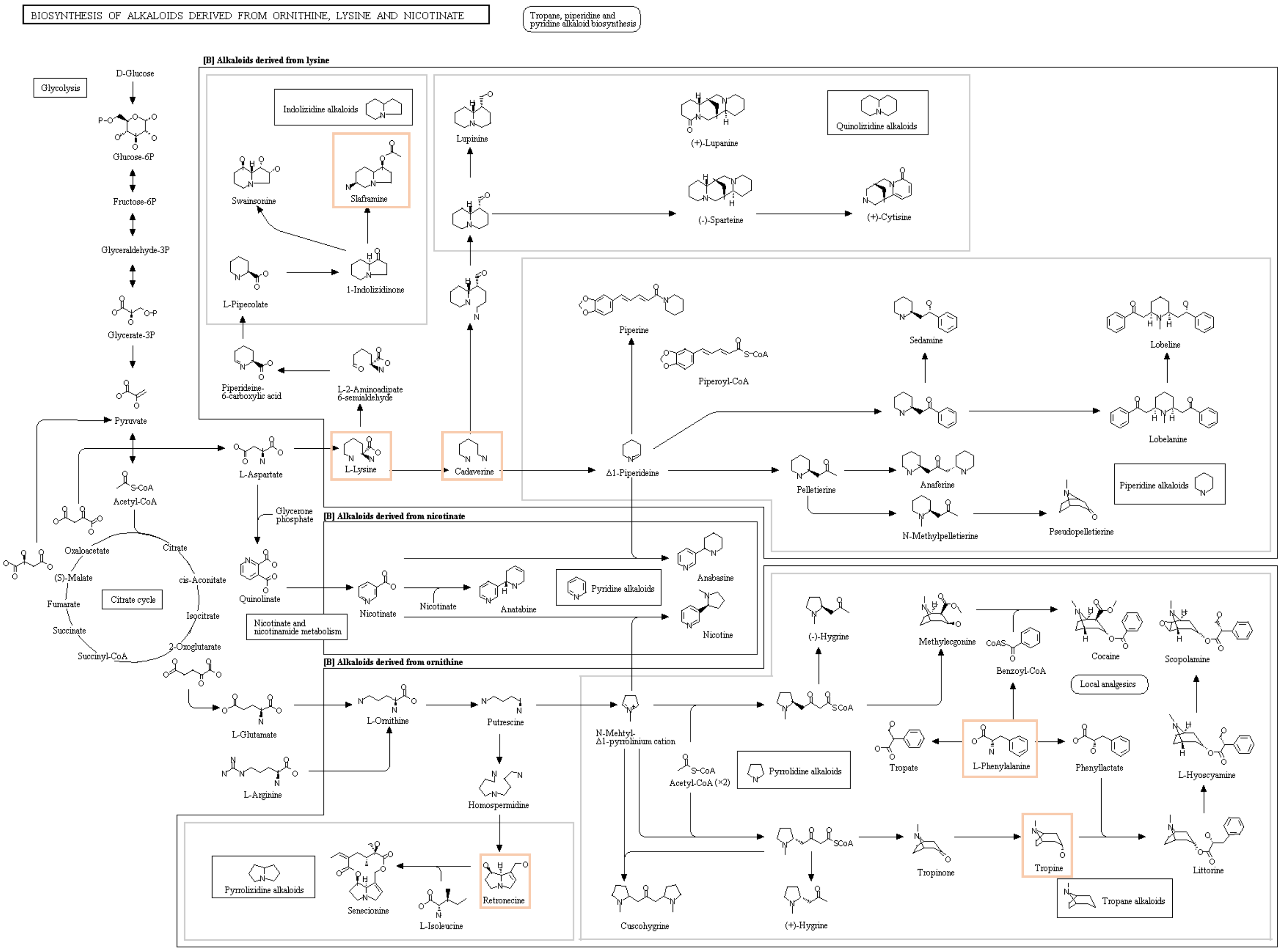

Figure S9. Differential mass features associated with DC lines mapped to biosynthesis of alkaloid derived from ornithine, lysine and nicotinic acid

Differential mass features mapped to biosynthesis of alkaloid derived from ornithine, lysine and nicotinic acid are shown in red, including L-lysine, cadaverine, slaframine, retronecine, L-phenylalanine and tropine.
