# Supplementary material for: Metabolic diversification of nitrogen‐containing metabolites by the expression of a heterologous lysine decarboxylase gene in Arabidopsis
Source: Plant J. 2019 Aug 27;100(3):505–21. doi: 10.1111/tpj.14454 (PMC6899585; doi:10.1111/tpj.14454)
Supplement: Supplementary file 11 — Figure S11. Identification of cadaverine in DC lines. [file TPJ-100-505-s011.pdf]

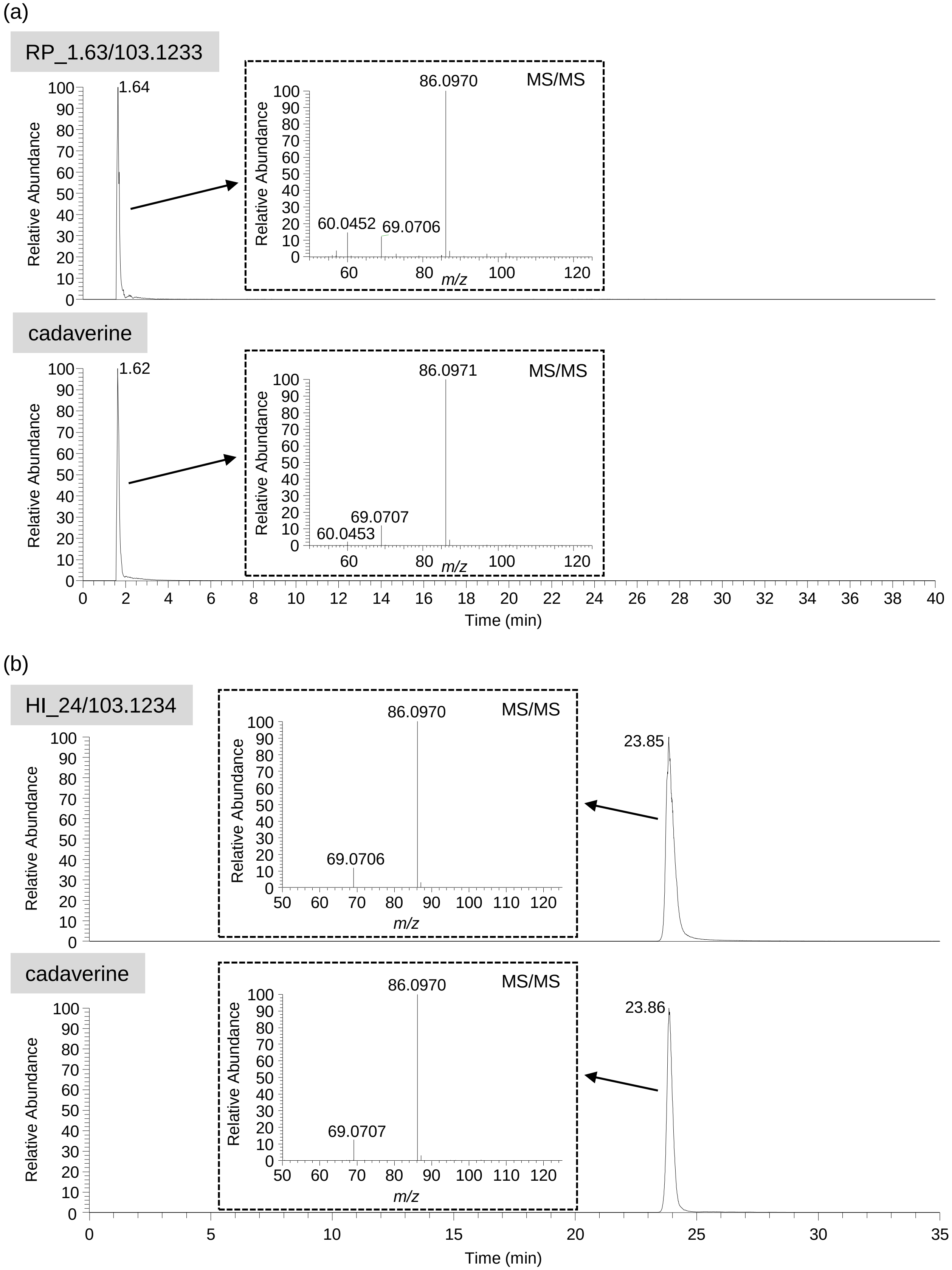


**Figure S11. Identification of cadaverine in DC lines**

Peaks of cadaverine in both (a) RPLC-mode and (b) HILIC-mode were identified with a metabolite standard. The retention times and MS/MS spectra for specific metabolite peaks were compared with those of cadaverine by LC-MS. Higher energy collision dissociation (HCD) mode was used to obtain MS/MS fragmentation.
