# Supplementary material for: Metabolic diversification of nitrogen‐containing metabolites by the expression of a heterologous lysine decarboxylase gene in Arabidopsis
Source: Plant J. 2019 Aug 27;100(3):505–21. doi: 10.1111/tpj.14454 (PMC6899585; doi:10.1111/tpj.14454)
Supplement: Supplementary file 12 — Figure S12. Enzymatic conversion of 5‐aminopentanal to 5‐aminopentanoate by AtALDH10A8 and AtALDH10A9. [file TPJ-100-505-s012.pdf]

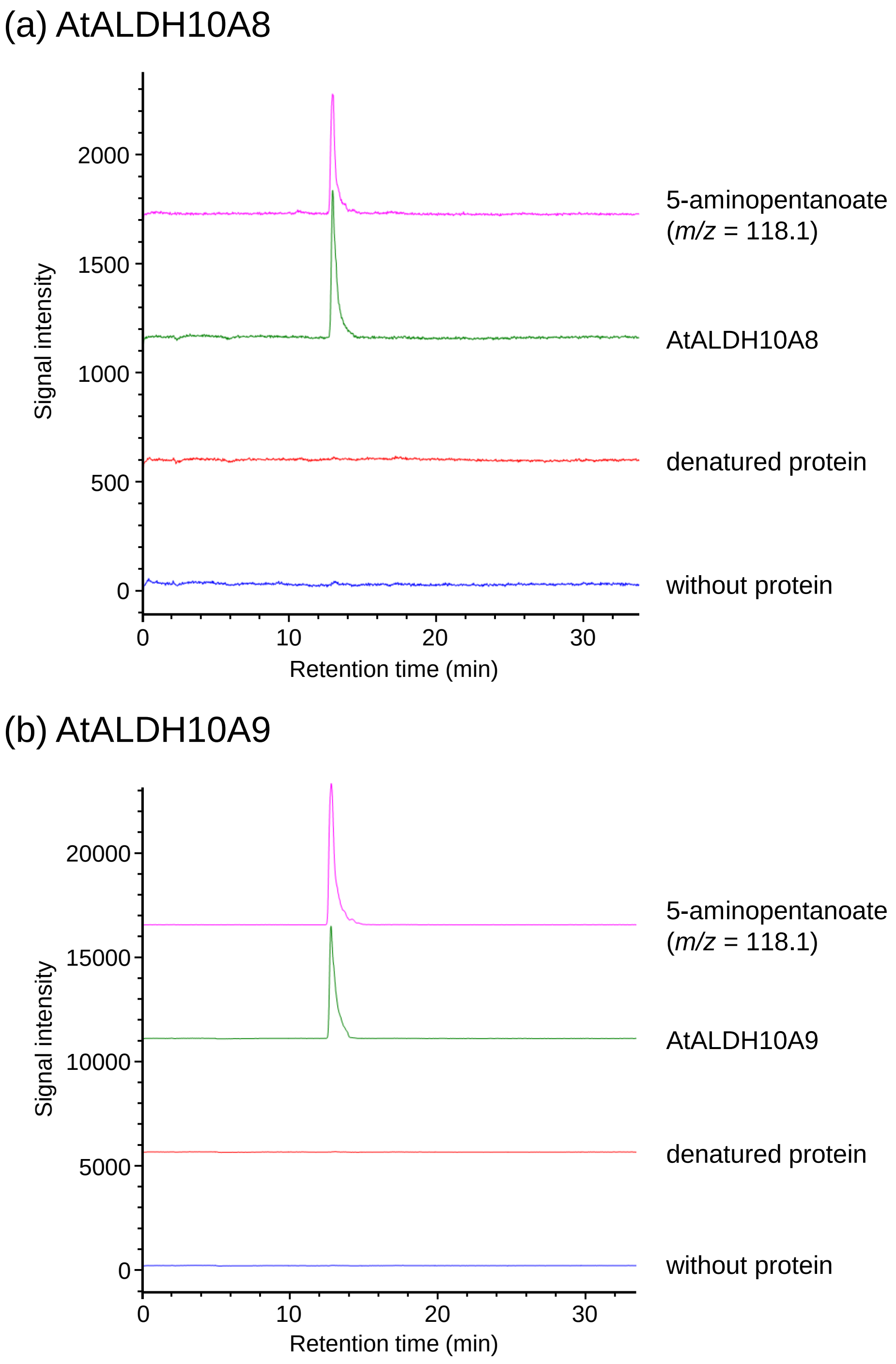

Figure S12. Enzymatic conversion of 5-aminopentanal to 5-aminopentanoate by AtALDH10A8 and AtALDH10A9

In vitro enzymatic assays of (a) AtALDH10A8 and (b) AtALDH10A9. The reaction product (5-aminopentanoate, *m/z* = 118.1) was monitored by single ion monitoring mode with LC-MS. 5-Aminopentanal was incubated with either a native recombinant enzyme, a heat-denatured enzyme or without protein. The 5-aminopentanoate was detected in both samples incubated with AtALDH10A8 and AtALDH10A9.
