# Supplementary material for: Metabolic diversification of nitrogen‐containing metabolites by the expression of a heterologous lysine decarboxylase gene in Arabidopsis
Source: Plant J. 2019 Aug 27;100(3):505–21. doi: 10.1111/tpj.14454 (PMC6899585; doi:10.1111/tpj.14454)
Supplement: Supplementary file 13 — Figure S13. Expression analysis for candidate genes associated with cadaverine catabolism. [file TPJ-100-505-s013.pdf]

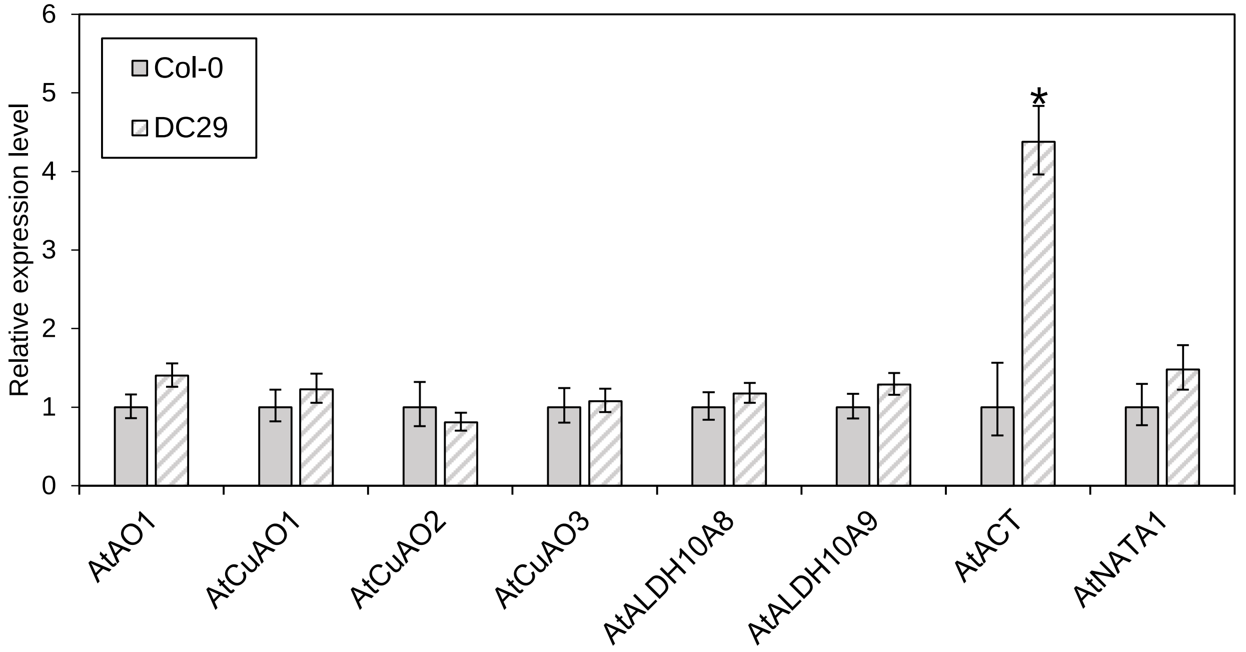

Figure S13. Expression analysis for candidate genes associated with cadaverine catabolism

Eight genes were selected based on their catalytic functions in catabolism of putrescine, an analog compound of cadaverine. Total RNA was extracted from two-week-old seedlings (30 seedlings were pooled and treated as a single biological replicate). Gene-specific primers were used for quantitative RT-PCR analysis. *β-Tubulin* was used as internal control. *AtAO*, *amine oxidase1*; *AtCuAO1-3*, *copper-containing amine oxidase1-3*; *AtALDH*, *aldehyde dehydrogenase*; *AtACT*, *agmatine coumaroyltransferase*; *AtNATA1*, *N-acetyltransferase activity1*. Data represent the mean ± standard error (biological replicate n = 4-5). *P < 0.05 (Student's t-test).
