# Supplementary material for: Metabolic diversification of nitrogen‐containing metabolites by the expression of a heterologous lysine decarboxylase gene in Arabidopsis
Source: Plant J. 2019 Aug 27;100(3):505–21. doi: 10.1111/tpj.14454 (PMC6899585; doi:10.1111/tpj.14454)
Supplement: Supplementary file 14 — Figure S14. Putrescine metabolism in Arabidopsis. [file TPJ-100-505-s014.pdf]

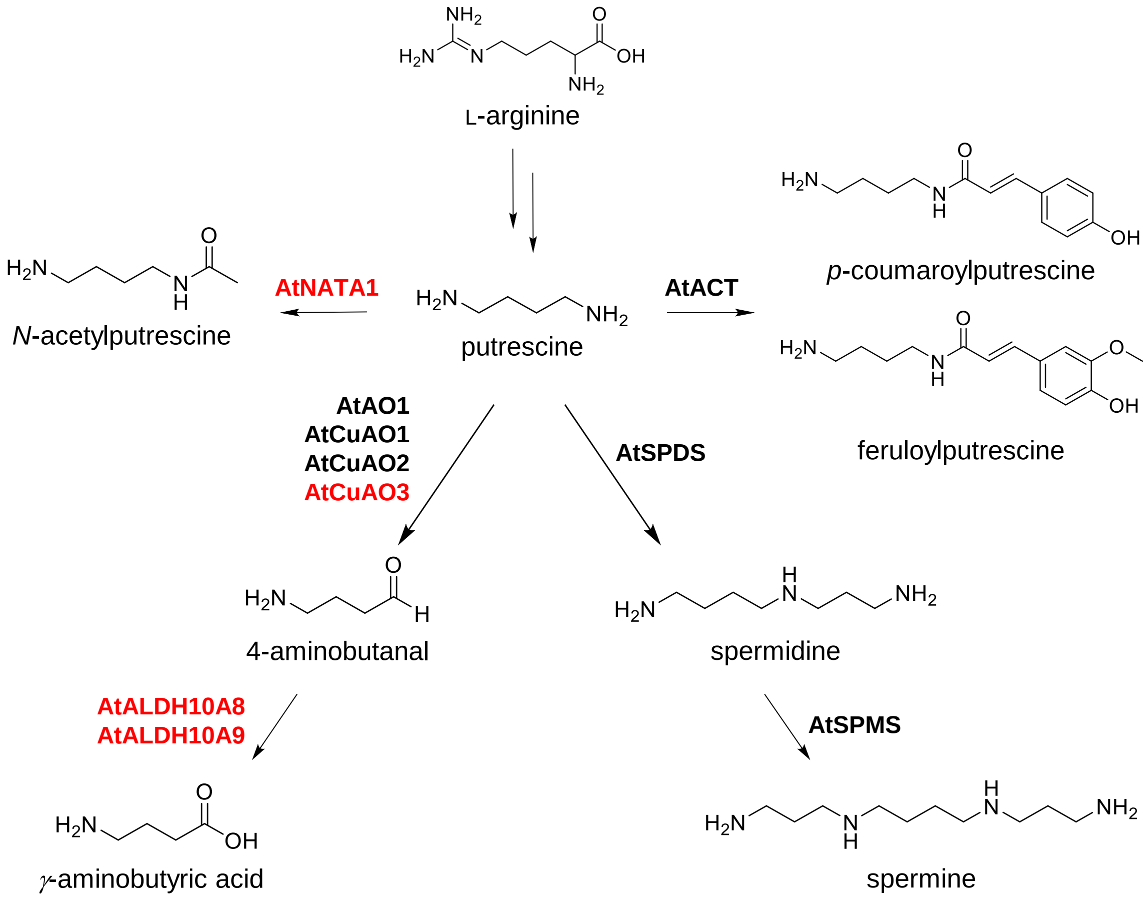

Figure S14. Putrescine metabolism in Arabidopsis
AtAO, amine oxidase; AtCuAO, copper-containing amine oxidase; AtALDH, aldehyde dehydrogenase; AtACT, agmatine coumaroyltransferase; AtSPDS, spermidine synthase; AtSPMS, spermine synthase; AtNATA1, *N*-acetyltransferase activity1. Enzymes possessing catalytic activity toward cadaverine or cadaverine catabolites are shown in red.
