# Supplementary material for: Metabolic diversification of nitrogen‐containing metabolites by the expression of a heterologous lysine decarboxylase gene in Arabidopsis
Source: Plant J. 2019 Aug 27;100(3):505–21. doi: 10.1111/tpj.14454 (PMC6899585; doi:10.1111/tpj.14454)
Supplement: Supplementary file 16 — Figure S16. Gene gain, loss, expansion and contraction of candidate genes coding enzymes associated with cadaverine catabolism across nine plant species. [file TPJ-100-505-s016.pdf]

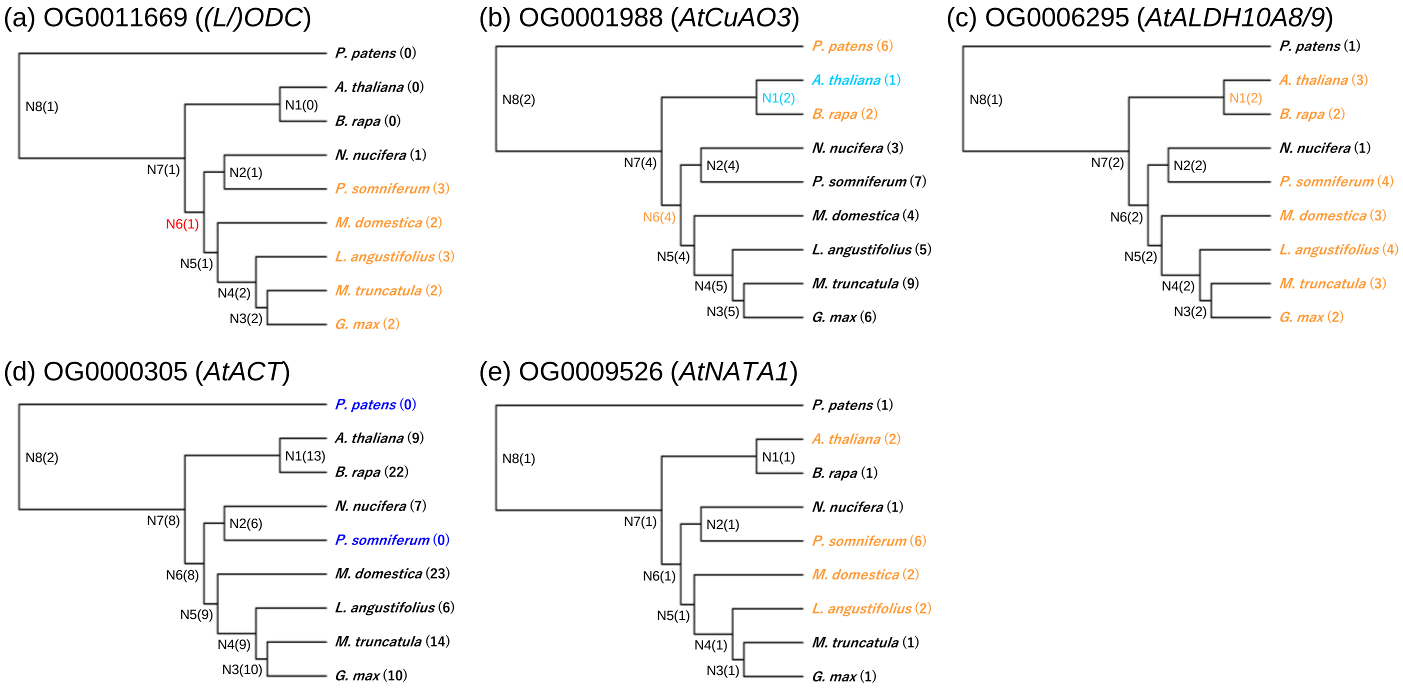

Figure S16. Gene gain, loss, expansion and contraction of candidate genes coding enzymes associated with cadaverine catabolism across nine plant species

Species trees with number of orthologs of candidate genes associated with cadaverine catabolism across nine plant species, including three ODC-lacking plants (*Physcomitrella patens*, *Arabidopsis thaliana* and *Brassica rapa*), two ODC-type plants (*Papaver somniferum* and *Malus domestica*) and four L/ODC-type plants (*Nelumbo nucifera*, *Lupinus angustifolius*, *Medicago truncatula* and *Glycine max*). Orthogroups of candidate genes including (a) OG0011669 ((*L/)ODC*), (b) OG0001988 (*AtCuAO3*), (c) OG0006295 (*AtALDH10A8/9*), (d) OG0000305 (*AtACT*) and (e) OG0009526 (*AtNATA1*) were obtained using OrthoFinder v2.31. The probability of gene gain, loss, expansion and contraction in each orthogroup were calculated by Count package. The nodes and taxons with high probability (>0.25) of gene gain, expansion, contraction and loss are shown in red, orange, sky blue and blue, respectively.
