# Supplementary material for: Metabolic diversification of nitrogen‐containing metabolites by the expression of a heterologous lysine decarboxylase gene in Arabidopsis
Source: Plant J. 2019 Aug 27;100(3):505–21. doi: 10.1111/tpj.14454 (PMC6899585; doi:10.1111/tpj.14454)
Supplement: Supplementary file 25 [file TPJ-100-505-s025.docx]

# Full legends for supporting information

**Figure S1. Structure of binary vector and semi-quantitative reverse transcription-PCR analysis of DC lines**

(a) Schematic diagram of the T-DNA region of the binary vector used to generate transgenic plants. (b) Gel images of semi-quantitative RT-PCR products of *La-L/ODC* and *β-tubulin* in 10-independent T_3_ transgenic lines. Thirty seedlings grown for two weeks were pooled and used for extraction of mRNA. Arabidopsis *β-tubulin* gene was used as an internal control. Based on band intensities, DC29, DC21 and DC42 were selected for further analysis. LB, left border; RB, right border; *P_35S_*, *cauliflower mosaic virus 35S promoter*; *E_Ω_*, *Ω enhancer*; *La-L/ODC*, *Lupinus angustifolius-lysine/ornithine decarboxylase*; *Th*, *heat shock protein terminator*; *Tn*, *nopaline synthase terminator*; *HPT*, *hygromycin phosphotransferase*.

**Figure S2. Accumulation levels of l-ornithine and putrescine in two-week-old seedlings**

(a) l-Ornithine and (b) putrescine levels in metabolites extracted from two-week-old seedlings (30 seedlings were pooled and treated as one biological replicate) were quantified with LC-MS. Data are the mean ± standard error (*n* = 4-6). ***P* < 0.01 (Student’s t-test). FW, fresh weight.

**Figure S3. Root length and biomass of DC lines**

(a) Comparison of root length of two-week-old DC lines. Values are means ± standard deviation (*n* = 6). (b) Biomass of seedlings grown for two weeks. Pooled two-week-old seedlings originating from 30 plants were regarded as one biological replicate for each line. Values are means ± standard deviation (*n* = 5). **P* < 0.05, ***P* < 0.01 (Student’s t-test). FW, fresh weight.

**Figure S4. Experimental workflow**

**Figure S5. R2 and Q2 values for OPLS-DA model**

Bar plots showing the R2 cumulative (green bar) representing the estimated fraction of total variance explained by the model, whereas Q2 cumulative (blue bar) indicates the accuracy of the model for prediction of variances. (a) RPLC-mode and (b) HILIC-mode.

**Figure S6. Differential mass features associated with DC lines mapped to arginine and proline metabolism**

Differential metabolite features mapped to arginine and proline metabolism are shown in red, including putrescine, *p*-coumaroylputrescine, *N*-acetylputrescine, *γ*-l-glutamylputrescine, spermidine, *N^2^*-succinyl-l-ornithine, *N*-carbamoylputrescine, agmatine, 5-aminopentanoate, 2-oxo-4-hydroxy-5-aminovalerate and 1-pyrroline-4-hydroxy-2-carboxylate.

**Figure S7. Differential mass features associated with DC lines mapped to phenylpropanoid biosynthesis**

Differential mass features mapped to phenylpropanoid biosynthesis are shown in red, including phenylalanine, *p*-coumaroylshikimic acid, ferulic acid, sinapic acid, sinapaldehyde, sinapyl alcohol, syringin and spermidine.

**Figure S8. Differential mass features in DC lines mapped to tropine, piperidine and pyridine alkaloid biosynthesis**

Differential mass features mapped to tropine, piperidine and pyridine alkaloid biosynthesis are shown in red, including cadaverine, 5-aminopentanal, slaframine, putrescine, retrotecine, tropine, l-phenylalanine and ecgonine.

**Figure S9. Differential mass features associated with DC lines mapped to biosynthesis of alkaloid derived from ornithine, lysine and nicotinic acid**

Differential mass features mapped to biosynthesis of alkaloid derived from ornithine, lysine and nicotinic acid are shown in red, including l-lysine, cadaverine, slaframine, retronecine, l-phenylalanine and tropine.

**Figure S10. Differential mass features associated with Col-0 mapped to lysine degradation pathway**

Differential mass features mapped to lysine degradation pathway are shown in red, including l-lysine, saccharopine and l-pipecolate.

**Figure S11. Identification of cadaverine in DC lines**

Peaks of cadaverine in both (a) RPLC-mode and (b) HILIC-mode were identified with a metabolite standard. The retention times and MS/MS spectra for specific metabolite peaks were compared with those of cadaverine by LC-MS. Higher energy collision dissociation (HCD) mode was used to obtain MS/MS fragmentation.

**Figure S12. Enzymatic conversion of 5-aminopentanal to 5-aminopentanoate by AtALDH10A8 and AtALDH10A9**

*In vitro* enzymatic assays of (a) AtALDH10A8 and (b) AtALDH10A9. The reaction product (5-aminopentanoate, *m/z* = 118.1) was monitored by single ion monitoring mode with LC-MS. 5-Aminopentanal was incubated with either a native recombinant enzyme, a heat-denatured enzyme or without protein. The 5-aminopentanoate was detected in both samples incubated with AtALDH10A8 and AtALDH10A9.

**Figure S13. Expression analysis for candidate genes associated with cadaverine catabolism**

Eight genes were selected based on their catalytic functions in catabolism of putrescine, an analog compound of cadaverine. Total RNA was extracted from two-week-old seedlings (30 seedlings were pooled and treated as a single biological replicate). Gene-specific primers were used for quantitative RT-PCR analysis. *β-Tubulin* was used as internal control. *AtAO*, *amine oxidase1*; *AtCuAO1-3*, *copper-containing amine oxidase1-3*; *AtALDH*, *aldehyde dehydrogenase*; *AtACT*, *agmatine coumaroyltransferase*; *AtNATA1*, *N-acetyltransferase activity1*. Data represent the mean ± standard error (biological replicate *n* = 4-5). **P* < 0.05 (Student’s t-test).

**Figure S14. Putrescine metabolism in Arabidopsis**

AtAO, amine oxidase; AtCuAO, copper-containing amine oxidase; AtALDH, aldehyde dehydrogenase; AtACT, agmatine coumaroyltransferase; AtSPDS, spermidine synthase; AtSPMS, spermine synthase; AtNATA1, *N*-acetyltransferase activity1. Enzymes possessing catalytic activity toward cadaverine or cadaverine catabolites are shown in red.

**Figure S15. Phylogenetic relationship of plant amine oxidases**

Each protein sequence in the tree is identified by its accession number, the respective plant species and the enzyme name (if available). Amino acid sequences were aligned with ClustalW and phylogenetic tree was generated using the Neighbor-Joining method with MEGA7. The bootstrap values obtained with 1000 replicates are shown next to the branches. The evolutionary distances were computed using the Poisson correction method. Red triangles indicate CuAO or CuAO-like enzymes in Arabidopsis and green boxes indicate enzymes shown to accept cadaverine as a substrate. Abbreviation: Ath, *Arabidopsis thaliana*; Atr, *Amborella trichopoda*; Bju, *Brassica juncea*; Car, *Cicer arietinum*; Ech, *Euphorbia characias*; Gma, *Glycine max*; Hvu, *Hordeum vulgare*; Lcu, *Lens culinaris*; Mdo, *Malus domestica*; Mtr, *Medicago truncatula*; Nta, *Nicotiana tabacum*; Osa, *Oryza sativa*; Psa, *Pisum sativum*; Psy, *Pinus sylvestris*; Ptr, *Populus trichocarpa*; Rco, *Ricinus communis*; Sbi, *Sorghum bicolor*; Sly, *Solanum lycopersicum*; Vvi, *Vitis vinifera*; Zma, *Zea mays.*

**Figure S16. Gene gain, loss, expansion and contraction of candidate genes coding enzymes associated with cadaverine catabolism across nine plant species**

Species trees with number of orthologs of candidate genes associated with cadaverine catabolism across nine plant species, including three ODC-lacking plants (*Physcomitrella patens*, *Arabidopsis thaliana* and *Brassica rapa*), two ODC-type plants (*Papaver somniferum* and *Malus domestica*) and four L/ODC-type plants (*Nelumbo nucifera*, *Lupinus angustifolius*, *Medicago truncatula* and *Glycine max*). Orthogroups of candidate genes including (a) OG0011669 (*(L/)ODC*), (b) OG0001988 (*AtCuAO3*), (c) OG0006295 (*AtALDH10A8/9*), (d) OG0000305 (*AtACT*) and (e) OG0009526 (*AtNATA1*) were obtained using OrthoFinder v2.31. The probability of gene gain, loss, expansion and contraction in each orthogroup were calculated by Count package. The nodes and taxons with high probability (>0.25) of gene gain, expansion, contraction and loss are shown in red, orange, sky blue and blue, respectively.

**Table S1. All detected peaks**

**Table S2. Results of OPLS-DA**

**Table S3. Differential mass features in DC lines and Col-0**

**Table S4. KEGG compound annotation for differential mass features**

**Table S5. Annotation and labeling ratios for specific peaks in DC lines**

**Table S6. Probability of gene gain, loss, expansion and contraction of candidate genes across nine plant species**

**Table S7. Primers used in this study**

**Table S8. Accession IDs of NCBI genome used in this study**
