# Supplementary material for: Metabolic diversification of nitrogen‐containing metabolites by the expression of a heterologous lysine decarboxylase gene in Arabidopsis
Source: Plant J. 2019 Aug 27;100(3):505–21. doi: 10.1111/tpj.14454 (PMC6899585; doi:10.1111/tpj.14454)
Supplement: Supplementary file 15 — Figure S15. Phylogenetic relationship of plant amine oxidases. [file TPJ-100-505-s015.pdf]

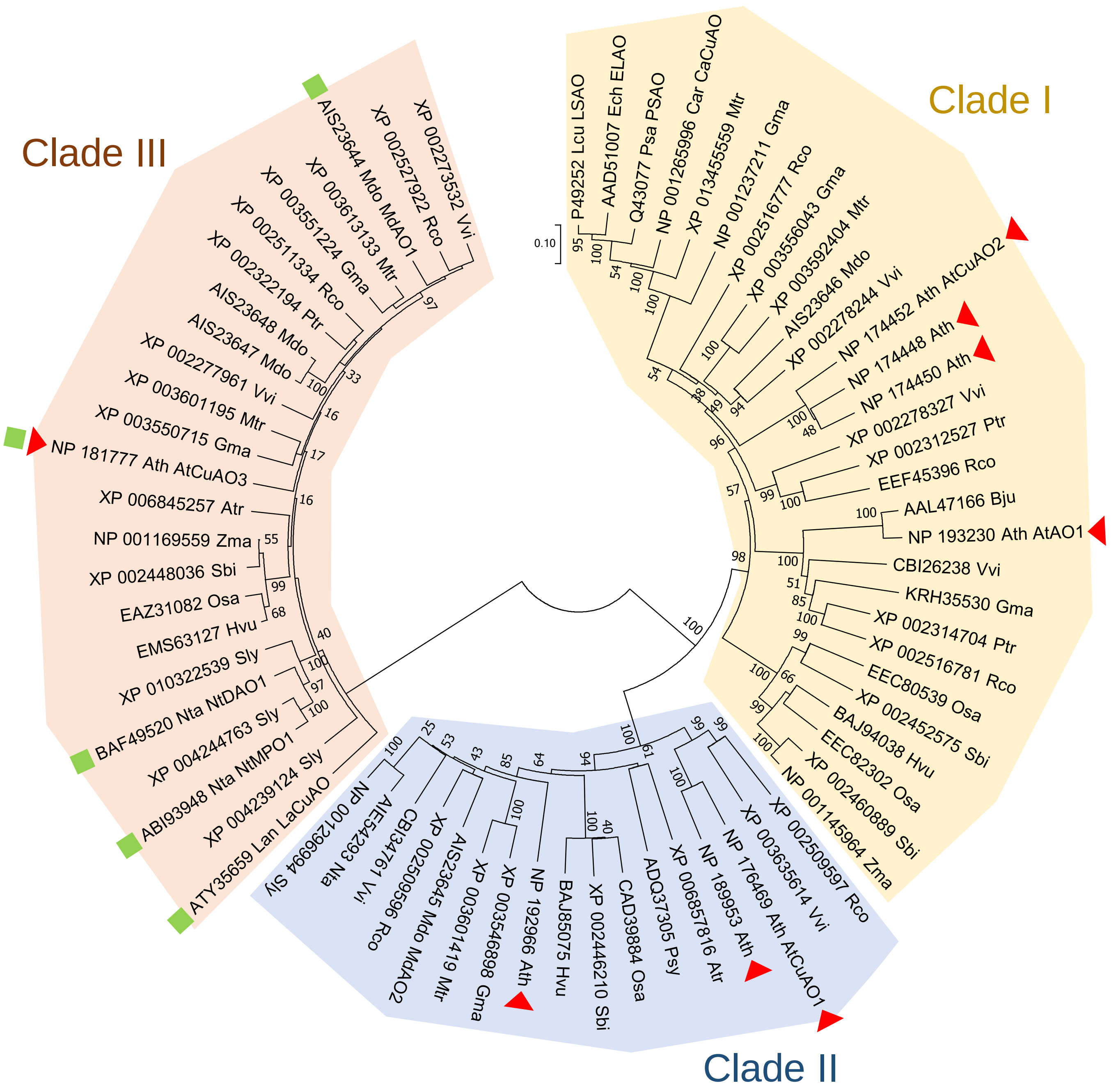

Figure S15. Phylogenetic relationship of plant amine oxidases
Each protein sequence in the tree is identified by its accession number, the respective plant species and the enzyme name (if available). Amino acid sequences were aligned with ClustalW and phylogenetic tree was generated using the Neighbor-Joining method with MEGA7. The bootstrap values obtained with 1000 replicates are shown next to the branches. The evolutionary distances were computed using the Poisson correction method. Red triangles indicate CuAO or CuAO-like enzymes in Arabidopsis and green boxes indicate enzymes shown to accept cadaverine as a substrate. Abbreviation: Ath, *Arabidopsis thaliana*; Atr, *Amborella trichopoda*; Bju, *Brassica juncea*; Car, *Cicer arietinum*; Ech, *Euphorbia characias*; Gma, *Glycine max*; Hvu, *Hordeum vulgare*; Lcu, *Lens culinaris*; Mdo, *Malus domestica*; Mtr, *Medicago truncatula*; Nta, *Nicotiana tabacum*; Osa, *Oryza sativa*; Psa, *Pisum sativum*; Psy, *Pinus sylvestris*; Ptr, *Populus trichocarpa*; Rco, *Ricinus communis*; Sbi, *Sorghum bicolor*; Sly, *Solanum lycopersicum*; Vvi, *Vitis vinifera*; Zma, *Zea mays.*
